# Data-Driven Quantitative Intrinsic Hazard Criteria for Nanoproduct Development in a Safe-by-Design Paradigm: A Case Study of Silver Nanoforms

**DOI:** 10.1021/acsanm.3c00173

**Published:** 2023-02-16

**Authors:** Irini Furxhi, Rossella Bengalli, Giulia Motta, Paride Mantecca, Ozge Kose, Marie Carriere, Ehtsham Ul Haq, Charlie O’Mahony, Magda Blosi, Davide Gardini, Anna Costa

**Affiliations:** † Transgero Ltd, Limerick V42V384, Ireland; ‡ Department of Accounting and Finance, Kemmy Business School, University of Limerick, Limerick V94T9PX, Ireland; § Department of Earth and Environmental Sciences, University of Milano-Bicocca, Piazza della Scienza 1, Milano 20126, Italy; ∥ Univ. Grenoble Alpes, CEA, CNRS, Grenoble INP, IRIG, SYMMES, Grenoble 38000, France; ⊥ Department of Physics, and Bernal Institute, University of Limerick, Limerick V94TC9PX, Ireland; # Istituto di Scienza e Tecnologia dei Materiali Ceramici (CNR-ISTEC), Via Granarolo, 64, Faenza 48018, Ravenna, Italy

**Keywords:** safe and sustainable by design, nanoforms, nanoparticles, quantitative structure−activity relationship, machine learning, Bayes rules, intrinsic hazard criteria

## Abstract

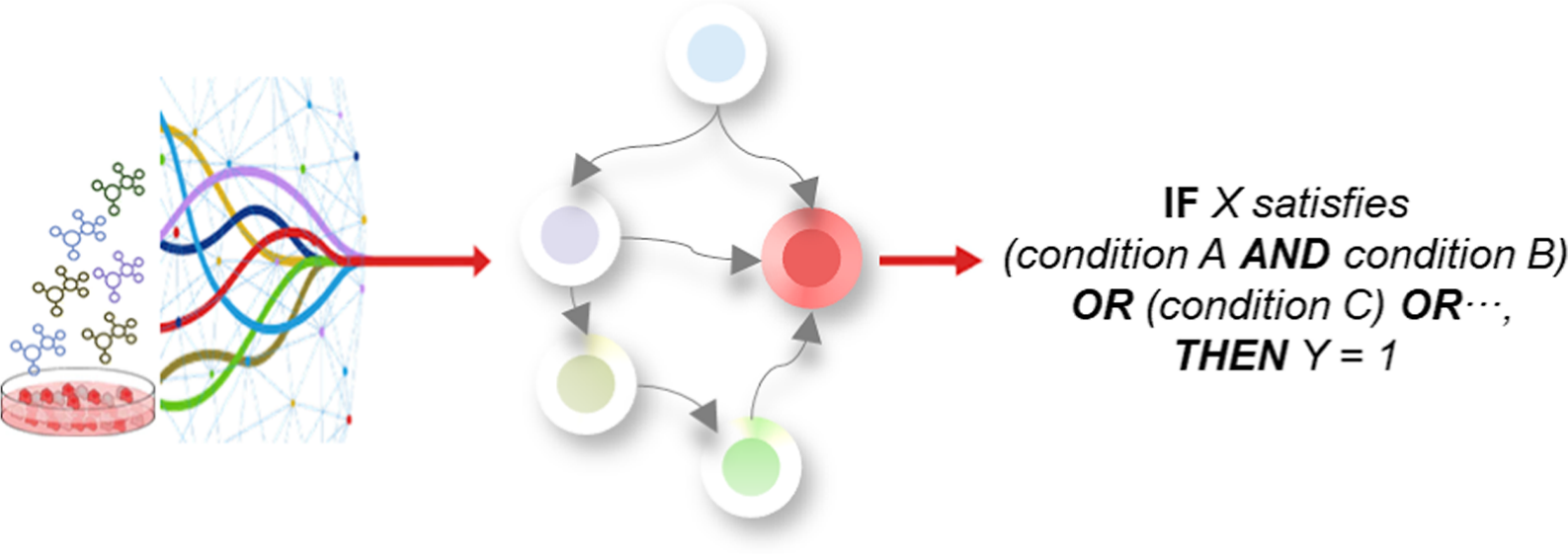

The current European (EU) policies, that is, the Green Deal, envisage safe and sustainable practices for chemicals, which include nanoforms (NFs), at the earliest stages of innovation. A theoretically safe and sustainable by design (SSbD) framework has been established from EU collaborative efforts toward the definition of quantitative criteria in each SSbD dimension, namely, the human and environmental safety dimension and the environmental, social, and economic sustainability dimensions. In this study, we target the safety dimension, and we demonstrate the journey toward quantitative intrinsic hazard criteria derived from findable, accessible, interoperable, and reusable data. Data were curated and merged for the development of new approach methodologies, that is, quantitative structure–activity relationship models based on regression and classification machine learning algorithms, with the intent to predict a hazard class. The models utilize system (i.e., hydrodynamic size and polydispersity index) and non-system (i.e., elemental composition and core size)-dependent nanoscale features in combination with biological in vitro attributes and experimental conditions for various silver NFs, functional antimicrobial textiles, and cosmetics applications. In a second step, interpretable rules (criteria) followed by a certainty factor were obtained by exploiting a Bayesian network structure crafted by expert reasoning. The probabilistic model shows a predictive capability of ≈78% (average accuracy across all hazard classes). In this work, we show how we shifted from the conceptualization of the SSbD framework toward the realistic implementation with pragmatic instances. This study reveals (i) quantitative intrinsic hazard criteria to be considered in the safety aspects during synthesis stage, (ii) the challenges within, and (iii) the future directions for the generation and distillation of such criteria that can feed SSbD paradigms. Specifically, the criteria can guide material engineers to synthesize NFs that are inherently safer from alternative nanoformulations, at the earliest stages of innovation, while the models enable a fast and cost-efficient in silico toxicological screening of previously synthesized and hypothetical scenarios of yet-to-be synthesized NFs.

## 1 Introduction

**Figure 1:**
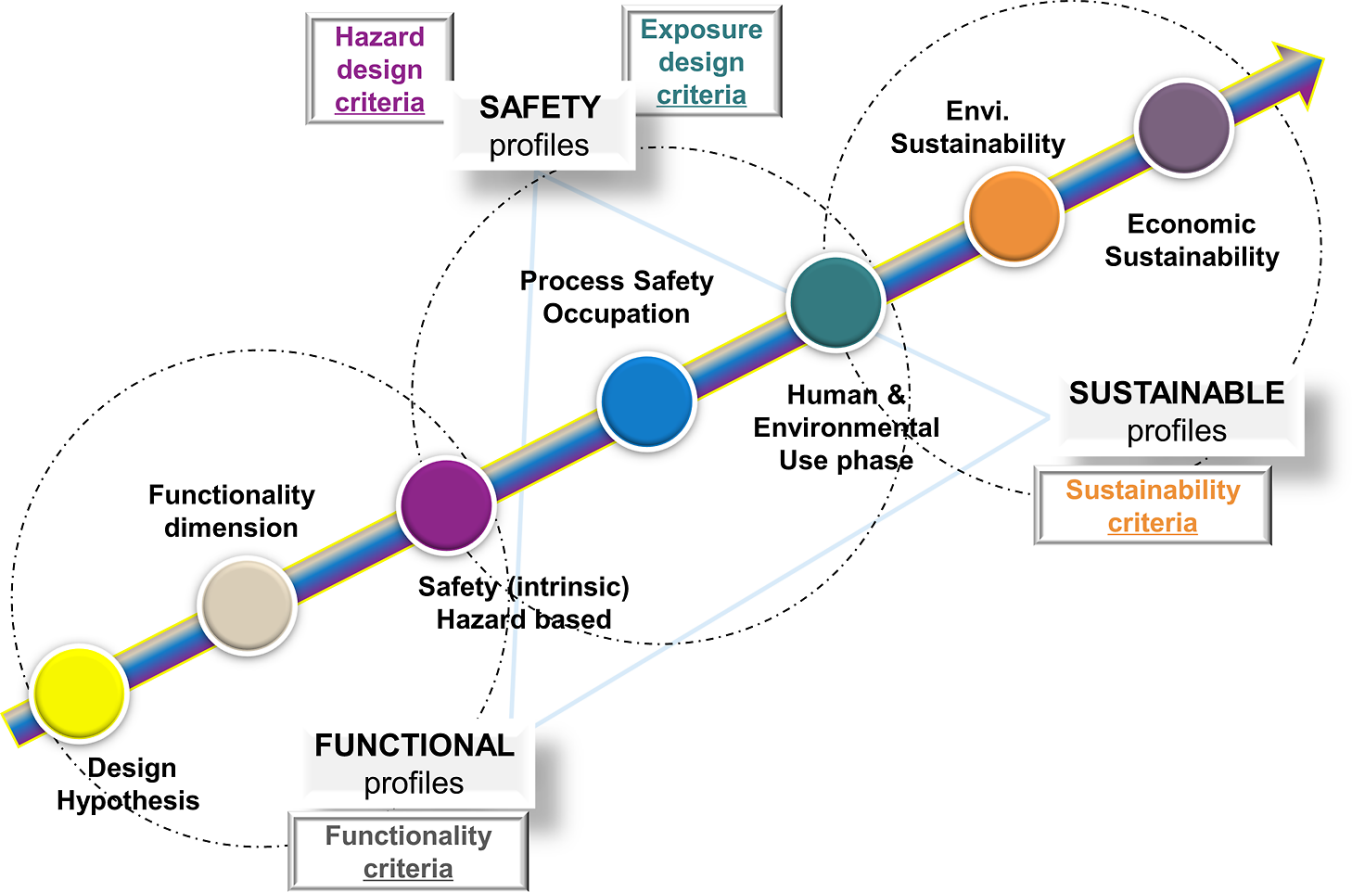
SSbD framework dimensions, following a hierarchical approach in which safety aspects are contemplated first, followed by environmental sustainability, and socioeconomic aspects (image adapted from the JRC framework).

The current paradigm of European (EU) policies, that is, the Green Deal, envisage safe and sustainable practices for chemicals, which include nanoforms (NFs), at the earliest stages of innovation to prevent and/or minimize safety and sustainability impacts.^1^ To meet those policy goals, novel frameworks are required such as the safe and sustainable by design (SSbD) notion. The SSbD concept is under the spotlight of science, regulation, and engineering to achieve the goals foreseen by EU policies.^2^ Commission has funded several projects on nanotechnologies^a^ in the frame of Horizon 2020 (H2020) which, through industrial case studies, will offer to various stakeholders digital products to facilitate (i) the selection of alternative design options and (ii) the decision-making process when having to weight criteria along the life cycle of a NF and once integrated in nano-enabled products (NEPs). In relation to the aforementioned criteria, the Joint Research Center (JRC) published a theoretical SSbD framework for the description of such criteria.^3^ The framework provides guiding principles on the SSbD dimensions to support the design phase and aspects and indicators in each dimension to establish criteria that will guide researchers toward SSbD practices. The SSbD dimensions are shown in Figure 1 demonstrating the re-design phase supported by a hypothesis formulation and the dimensional targets of functionality, human, and environmental safety containing intrinsic hazards, human occupational safety (process stage) and human/environmental health (use phase), and the two final steps of sustainability (environmental and economic).

The first principle stressed by ref (4) that supports the SSbD framework is the need of findable, accessible, interoperable, and reusable (FAIR) data: each dimension is driven by criteria based on data (experimental or modeled) to promote safe and sustainable research and innovation. A cornerstone aspect of the implementation and reproducibility of the SSbD concept is data quality and availability, that is, data FAIRness. Data needs to be treated according to FAIR principles to safeguard its long-term use and access.^5,6^ The data management plan has been added as an inherent deliverable of any project that generates, assembles, or processes data according to the guidelines on FAIR data management. The Anticipating Safety Issues at the Design Stage of NAno Product Development (ASINA) project is generating data across the life cycle of NFs with the aim to develop a data-driven decision-making strategy based on the manufacturing of two types of enhanced antimicrobial NEPs, namely, functional textiles and cosmetics applications.^7^ These data are currently being curated by the data shepherd^b^.^8−10^ In this study, we show one of the fruits of the FAIR data management process and how such an action accelerates the development of new approach methodologies (NAMs).

The second principle underlined by ref (4) is the need of NAMs: an umbrella of various applications such as computational, that is, in chemico, in silico, and other in vitro, approaches that allow multiple investigations at the same time and are expected to accelerate the implementation and validation of the SSbD concept^c^.^11−13^ Machine learning (ML) is a subfield of artificial intelligence (AI) and represents the definitive implementation of the 3R principles (replacement, reduction, and refinement of animal testing). In the field of computational (nano)toxicology, one of the most essential methods are the quantitative structure–activity relationships (QSARs, “nano-QSARs”, when applied to NFs). In QSAR, the activity (e.g., toxicity) is predicted from a set of descriptors by using various ML algorithms (e.g., supporting vector machines, random forests, and artificial neural networks).^14^ QSARs have been widely used in the field of nanotoxicology,^15−18^ and with the blooming of the ML applications, the interpretability has become an integral part so that their reasoning processes are more understandable and easier to be used in practice.^19^ Bayesian networks (BNs) are ML graphical models that merge probabilistic analysis, automated reasoning, and expert judgment. Such an amalgamation is essential in a challenging domain, such as nanosafety, which faces conflicting and uncertain knowledge.^20,21^ Expert reasoning structures are interpretable, re-usable by humans, and differ from the ones generated solely by automated reasoning from data.^22^ Numerous studies have employed BNs in the nanosafety domain to support risk assessment and prioritize NF hazard assessment.^16,23−27^

The third principle for a successful SSbD implementation is the extraction of quantitative criteria: during the EU high-level roundtable on chemicals strategy for sustainability,^c^ it was mentioned that the design criteria for chemicals will move “from qualitative to quantitative assessments, with more data becoming available”. Recent stakeholder webinars^d^, networking events^e^, and nanosafety expert trainings^f^ stressed that a well-defined and straightforward approach to derive quantitative criteria guiding a SSbD is missing and required. The BNs fulfill the expectations of such criteria by providing a set of discrete and mutually dependent interpretable rules from the conditional probability tables (CPTs). These rules have been used to guide decision-making processes by providing experts a series of IF statements [IF *X* satisfies (condition A AND condition B) OR (condition C) OR..., Then *Y* = 1].^28,29^ In the nanosafety domain,^30^ BNs were developed from the data derived from a meta-analysis of cellular viability of quantum dot NFs. In the supplementary material of the *ibid* study, the authors provide such rules derived from the CPTs of the BN structure.

**Figure 2:**
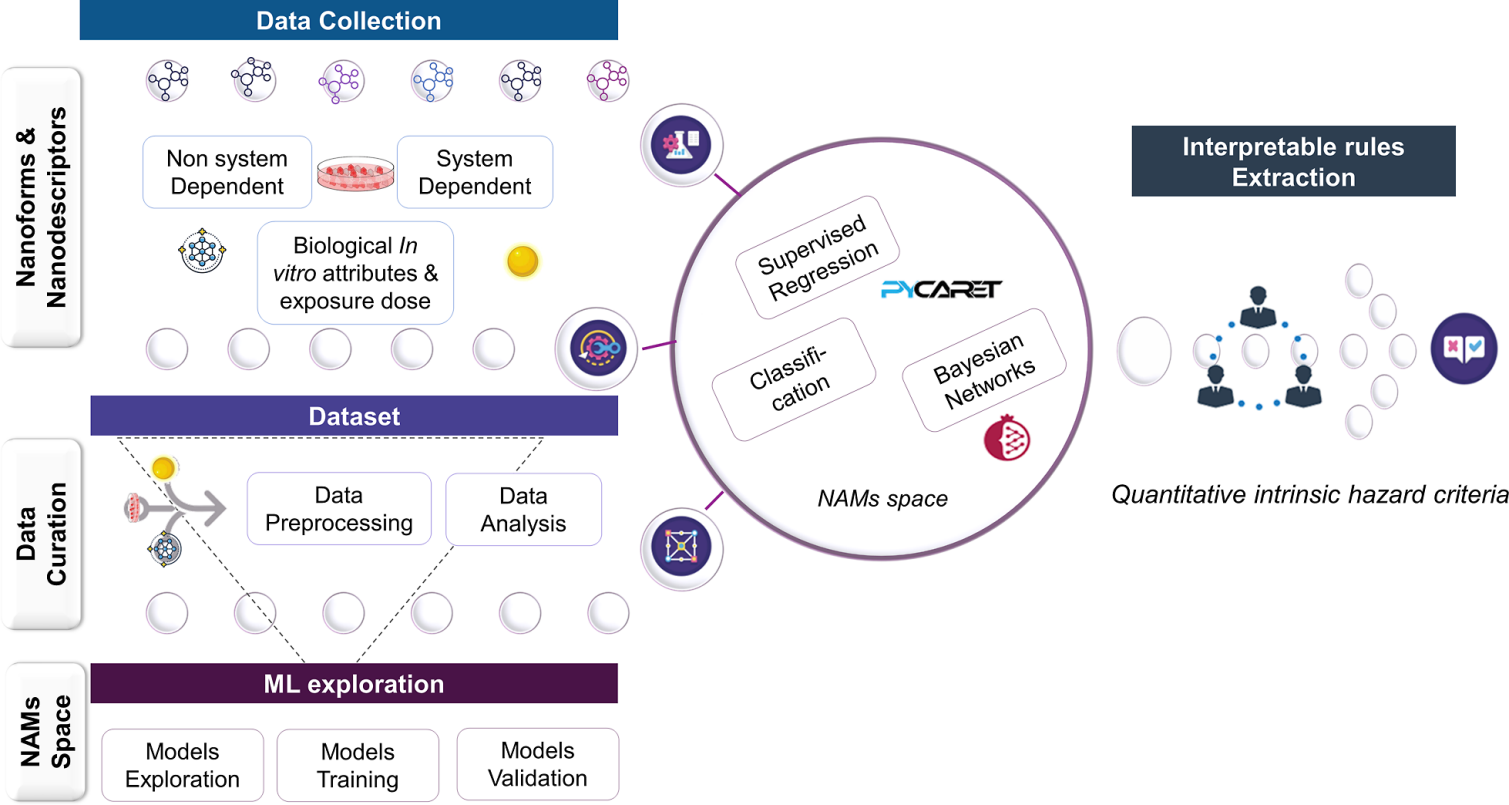
Interpretable rule extraction workflow. From the data collection, to dataset curation, and exploration of ML tools for the QSAR development to the final extrapolation of interpretable rules.

To respond to the above challenges, we focused on step 1—hazard assessment of the SSbD framework^3^ concerning the assessment of the physicochemical (pchem) properties of materials in order to derive criteria that lead to intrinsically safer materials, before proceeding further in the SSbD execution. In this context, the term “by design” refers to a set of nanoscale features that can be modified by material designers toward synthesizing/re-designing less hazardous NFs. In this manner, our efforts align with the overall objectives of the chemicals strategy for sustainability^g^, for example, “ensure that all materials placed on the market are in themselves safe”. The methodology followed is shown in Figure 2. We combined FAIR data with NAMs comprising QSAR approaches and explainable ML techniques for the extraction of interpretable rules from BNs. Data collection: first, nanodescriptors related to silver nanoforms (AgNFs) such as system-dependent pchem properties (extrinsic properties influenced by the surrounding environment or experimental conditions aka the system) and non-system-dependent pchem properties (intrinsic), biological in vitro attributes, exposure conditions, and the hazard outcome were collected (Figure 2, left). Dataset: in a second step, data is curated, merged, and processed with various techniques (such as missing value imputations and one hot encoding). Data is analyzed for visualization and insight purposes. ML explorations for QSAR development: various ML models were explored, from regression and classifications algorithms to the construction of a constrained BN based on expert judgment. Models are trained and validated to reveal predictive performance metrics. Rules extraction: finally, the quantitative intrinsic hazard criteria (rules) applicable to the safety dimension of the SSbD framework were extracted from the BN structure (Figure 2, right). From now on, in this manuscript, the interpretable rules refer to the quantitative intrinsic hazard criteria.

This work demonstrates for the first time how QSAR models in combination with FAIR data can support the development and implementation of SSbD paradigm by supporting the knowledge establishment for criteria definition.

## 2 Experimental Section

### 2.1 Data Collection

The data gathered for this study refers to AgNFs, which are currently under investigation as SSbD alternatives.^31,32^

#### 2.1.1 Silver NFs

Based on the intended application, data of two alternative AgNFs coated with hydroxyethylcellulose (HEC), either as powder form or suspended into a solution, are gathered. The powder is intended for incorporation into cosmetics to provide functionalities such as antimicrobial creams or lotions, while the solution is incorporated into textiles as coating for increased antimicrobial/antiviral efficiency.^33,34^ In addition to the alternative AgNFs, reference data are used to facilitate the NAM approach. Commission^4^ mentions the need of reference materials data for the validation of NAMs derived from harmonized protocols. Below, we describe the NFs along with their European Registry of Materials (ERM) identifiers which guarantee that internal documentation can be later connected to data and expertise for the particular NFs or variants^35^

(1) ERM00000559: AgHEC water-based solution (AgHEC sol) reduced from AgNO_3_ solution by HEC catalyzed by sodium hydroxide (NaOH). From a sustainability perception, the one-step synthesis process utilized is an environment-friendly, sol–gel-based technology obtainable at room temperature^h^.
(2) ERM00000552: Powder AgHEC (AgHEC pwd) is derived by spray-freeze-drying the solution without affecting the organic layer producing microparticles with highly porous nanostructures (final composition of 11% Ag and 89% wt HEC). Both solution and powder contain a molar ratio of HEC/Ag = 5.5 and NaOH/Ag = 2.8.
(3) ERM00000549: Reference uncoated material—Sigma-Aldrich (Ag ref).
(4) ERM00000548: Reference material coated with PVP—Sigma-Aldrich (AgPVP ref) in a powder form.
(5) ERM00000575: A variant AgHEC with a HEC/Ag: 6.4 and NaOH/Ag: 1.4 molar ratio (AgHEC 6.4/1.4 sol) as a SSbD alternative. Formulation obtained by tweaking two synthesis parameters that affect the antimicrobial effectiveness (i) concentration of HEC, acting as both reducing and chelating agents, and (ii) concentration of NaOH, acting as a catalyst. Such reagents play a fundamental role in the nucleation and growth processes, colloidal stability,^36^ and reduction of metals,^37^ driving the formation of the non-aggregated NFs.
(6) ERM00000580: AgNFs with curcumin solution (AgCUR) which is a proven antibacterial/antiviral phytocompound as a SSbD alternative.^38^

#### 2.1.2 Input Features

It is of fundamental importance to define the nanoscale features–nanodescriptors used in the QSAR modeling since a subtle alteration may influence the output to be predicted (i.e., cellular viability). Nanodescriptors should reflect not only the substance elemental composition but also other characteristics requested by regulation when reporting a NF, for example, size distribution and other morphological characterization such as crystal structure.^39^ Moreover, nanodescriptors should reflect the influence of the system (surrounding environment or experimental conditions) on those properties. One can differentiate between system-independent (intrinsic properties) and system-dependent (extrinsic properties) nanodescriptors.^40^ In this study, both are considered. It is worth to notice that the dataset solely refers to AgNFs, rendering those descriptors collectively unique, acting as a fingerprint. This enables NFs belonging in the same elemental composition group to be differentiated.^40^ Commission^4^ remarks the development of substance-specific hazard assessments and the exploration of the determinants that drive the toxicity.

##### 2.1.2.1 System-Independent Nanodescriptors

Those descriptors contain the (i) quantification of the atomic concentrations of elemental compositions derived by X-ray photoelectron spectroscopy (XPS) analysis, a technique for analyzing material’s surface chemistry,^41^ (ii) core size and morphology by transmission electron microscopy (TEM), and (3) crystallographic structure-related information by X-ray diffraction analysis (XRD).^42^ System-dependent nanodescriptors: a crucial aspect of NF toxicity exploration is their characterization in relevant biological media since properties could change in relation to the environment, influencing their cytotoxicity potential. Therefore, NFs should be characterized as pristine (system independent) and as applied in biological fluids,^43,44^ which in this case is the cell culture medium [Dulbecco’s modified Eagle’s medium (DMEM) and 1% fetal bovine serum (FBS) with pH = 7, 2–7, and 4]. The nanodescriptors contain particle’s hydrodynamic size (z-average and peak maximum value) and polydispersity indexes (PdI), which represent the sample’s heterogeneity, derived from dynamic light scattering (DLS). DLS analysis is recommended from the ISO standard^i^ and from the OECD’s Working Party on Manufactured Nanomaterials (WPMN) testing programme^j^. Since DLS measurements of size distribution depend on sample dispersion, PdI should be considered.^45^ PdI values vary from 0.01 to 0.5–0.7 (monodispersed particles) and PdI >0.7 (broad particle size distribution).^46^ Moreover, size distribution could change at time 0 (*t*_0_) and the time after in vitro exposure (in our case 24 h, for cell viability assessment, *t*_24_). To account for alterations of properties in time,^47^ we considered measurements (hydrodynamic size and PdIs) performed at *t*_0_ and *t*_24._

##### 2.1.2.2 Biological Attributes

The criteria definition in the SSbD framework was based on hazard categories established within the CLP [no. 1272/2008 (EU, 2008)] and REACH (no. 1907/2006 (EU, 2006)] regulations containing carcinogenicity, reproductive toxicity, target organ toxicity, and so forth. However, those endpoints are assessed with in vivo testing. In our case, the in vitro lines represent different target organs, alveolar lung cells (A549, human adenocarcinoma), and intestinal (HCT-116, human colon carcinoma) at a cellular level of biological representation. The cell lines represent different exposure routes, that is, inhalation and ingestion. Inhalation is a major route of human exposure to airborne NFs, and it may occur at workplaces, and A549 cells are a well-established line used for inhalation toxicological testing,^48^ including AgNFs.^49^ Ingestion is another important route of exposure,^50^ and with regard to intestinal exposure, ingested NFs pass through various environments before reaching the intestinal cells, such as saliva, gastric, and intestinal fluids.^51,52^ Due to the complex nature of these fluids such as acidic conditions, the presence of salts and biomolecules, the pchem properties of NFs could be altered before, during, and after passing the gastrointestinal tract, affecting their bioactivity.^53,54^ To mimic the fate of NFs, simulated digestive fluids were prepared based on ref (55), and NF preparation in simulated digestion cascade was performed according to ref (56). Finally, digested and non-digested NFs were exposed to HCT-116 cells, a well-accepted model for testing NF intestinal cytotoxicity.^57^

##### 2.1.2.3 Outcome and Exposure Conditions

Hazard evaluation was performed via cytotoxic measurements based on cell viability, a means to a preliminary hazard screening in a quick, cheap, and efficient manner.^58^ Several in vitro assays are available to assess cell viability, including the 3-(4,5-dimethylthiazol-2-yl)-2,5-diphenyltetrazolium bromide (MTT), Alamar blue, and WST-1 tests, which are rapid, high-throughput, and low-cost assays.^59^ The cell viability for the lung cells (%) was estimated with MTT and Alamar blue protocols (ISO10993-5:2009) at various concentrations (0.1–100 ppm). MTT is used in several ISO standards (ISO10993-5:2009) and OECD test guidelines (OECD 431, 439, 492). The inclusion of the different assays as output-related features is also relevant since NFs could interfere with the tests and the final outcome.^60,61^ Intestinal cells are exposed with either digested or non-digested AgNFs at concentrations ranging from 1.25 to 100 ppm with the WST-1 assay. All experiments refer to a 24 h duration of exposure.

### 2.2 Dataset

#### 2.2.1 Dataset Curation

The three different datasets are as follows: toxicological attributes in (i) lung and (ii) intestinal cell lines along with system-dependent features and (iii) system-independent pchem properties were merged. Each row represents one set of experimental testing conditions and related system-dependent nanodescriptors based on the exposure dose and NF pre-treatment (for intestinal assessments). The system-independent inputs are NF specific and independent of experimental conditions. Data is captured via FAIR principles where the reader can find the origin (institution) of each data, the responsible data creators (experimentalists), the raw measurements, the protocols followed, and the instrumentations used for each experiment. More information regarding the worksheet used for data capturing can be found here.^8^

#### 2.2.2 Data Preprocessing

Missing value imputation methodology is commonly used for ML studies since it is a basic assumption that (i) certain relationship exists between the different attributes and (ii) missing value fill-in is a learning process.^62^ Missing value imputation: for the missing values of system-dependent nanodescriptors, imputation was performed by linearly interpolating data in cases where the corresponding variable was known in a smaller and larger dose; for example, if the hydrodynamic size at *t*_0_ was known for a 10 and 50 ppm solution, interpolation for the 20 and 40 ppm solution was feasible (neighboring points according to the corresponding values). The cases above and below those known values were left blank. The missing value interpolation was performed on a dose, cell line, and NF’s pretreatment-reliant manner. Meaning, if the hydrodynamic size at a 50 ppm solution for one specific digested NF was known, the same value did not apply for the non-digested NFs at the same 50 ppm solution. In this manner, we kept the missing value imputation uncertainty at minimum levels. For the system-independent missing values and for the ones left blank from the interpolation, an iterative sequential imputation process was executed via regression with the Light Gradient Boosting Machine (lightgbm) algorithm.^63^ Each feature is modeled as a function of the other features, allowing prior values to be used into predicting subsequent features. The dataset with the ML-based imputed values can be found in the supplementary material (Supporting Information: tab v01 in the excel). It is worth to notice that during the ML imputation, the ERM codes of the NFs were left in the dataset (dropped after for modeling) since it is the only feature that distinguishes the dataset into fragments, greatly easing the lightgbm algorithm with targeted imputations, lowering the uncertainties.

##### 2.2.2.1 One Hot Encoding

One hot encoding was performed on the categorical attributes for the ML regression models and the BNs. This technique converts categorical features into numerical dummy variables with values 0/1 indicating the absence or presence of the originally feature.^64^

##### 2.2.2.2 SMOTE

For the ML classifiers and BNs, the outcome was discretized into three classes: safe, toxic, and very toxic depending on the corresponding values of cell viability. A challenge in the criteria development is the threshold definition for deciding when a material is deemed safe.^1^ The lower the viability value, the higher the cytotoxic potential. Thus, safe were the data points with cell viability ≥70%, toxic where the viability ranged from 30 to 70% in a precautionary manner, and very toxic where the viability was <30% (ISO10993-5). However, discretizing the outcome leads to unbalanced classes. To address this issue, we adjusted the relative frequency of the instances by applying SMOTE (synthetic minority oversampling technique), a supervised algorithm that uses the *k*-nearest neighbors algorithm in the training set (80%) to oversample minority instances.^65^

##### 2.2.2.3 Discretization

In the case of BNs, a quantile-based discretization function was performed on the numerical inputs to discretize them into three equal-sized bins to facilitate the interpretation of the rules. Instead of utilizing the actual numeric edges of the bins, the function defines the bins using percentiles based on the data distribution.

#### 2.2.3 Data Analysis and Visualization

##### 2.2.3.1 UMAP and MAPPER

Uniform manifold approximation and projection (UMAP), like principal component analysis methodology, is a dimension reduction technique for 3D data structure visualization. UMAP is constructed from a theoretical framework based in Riemannian geometry and algebraic topology.^66^ Prior to patching together their local fuzzy simplicial set representations, it first builds a topological representation of the high-dimensional data with local manifold approximations.^67^ Similarly, the Mapper algorithm was used for visualization purposes, a method for extracting simple descriptions of the dataset in the forms of simplicial complexes.^68^ The methodology is qualitative based on topological ideas and on partial clustering guided by a set of functions defined on the min–max scaled data. Mapper is essentially providing a simplified version of the UMAP scatterplot via topology.^69^

##### 2.2.3.2 Correlation

Spearman’s was performed on numerical–numerical correlations which ranks correlation coefficient (ρ) as a measure of monotonic correlation between −1 and +1, where −1 indicates the negative correlation, 0 denotes the absence of association, and 1 shows the positive correlation.^70^ Cramér’s V, an association measure for categorical variables,^71^ was utilized for numerical–categorical and categorical–categorical features with coefficients ranging from 0 to 1, with 0 denoting independence and 1 indicating perfect correlation.

### 2.3 QSAR Development

#### 2.3.1 ML Exploration

Several QSAR models were developed exploring various ML algorithms via PyCaret, a low-code AutoML-augmented Data Pipeline library implemented in Python version 3.7.^72^ Regression algorithms include lightgbm, random forest regressor (rfr), extra trees regressor (etr), Lasso regression (lasso), elastic net (en), linear regression (lr), AdaBoost regressor (ada), and so forth; classification algorithms include gradient boosting classifier (gbc), random forest classifier (rf), extra trees classifier (et), decision tree classifier (dt), ridge classifier (ridge), linear discriminant analysis (lda), and so forth. All models are trained with a randomly split sample containing 80% of the initial dataset, with 20% withheld for an out-of-sample validation.

#### 2.3.2 BNs and Rules Extraction

BNs are directed acyclic graphical models where features are nodes and connections are arrows, each of which denotes a conditional reliance of a child to a parent node. The Bayes’ rule updates the probabilities in light of new data, and the network as a whole represents the joint probability distribution of features.^73−75^ The probability distribution of all nodes is specified by the artifact of all CPTs in the BN model. For the development of the BN structure and the CPTs, we utilized an open source ML package for probabilistic modeling in Python, pomegranate.^76^ We initialized the BN structure development in a two-fold manner. First, the optimal unconstrained structure was built on the basis of the exact algorithm with knowledge learned directly from data without interference. Second, the structure was then refined by guidance upon expert judgment and inclusion of enforced expected dependencies.^77^ The BN constructed in this manner encodes the expert’s reasoning process and allows the system to explain the inference through interpretable rules.^22,78^

Structure learning and rules extraction are independent with the latter being described as an explainability method.^77^ Each rule is followed with a certainty factor (CF), which is the likelihood ratio for and against an outcome (*T*) when presented with evidence (*X*): that is, IF (*X* = 0) THEN *T* = 0 with CF = 0.25. By adding CF to rules, we reveal model’s uncertainty in the nanosafety domain.^22^

#### 2.3.3 Models Validation

QSAR models were validated based on the OECD guiding principles, containing a defined endpoint (biological effect can be measured and modeled, i.e., cellular viability); unambiguous algorithms and measurements of goodness-of-fit, robustness (internal 10-fold cross-validation, 80%), and predictivity (external validation, 20%).^79,80^ Since the focus of this study is QSAR based on the BN algorithm, the domain of applicability is defined solely for this case. Regression QSAR models were validated with various performance metrics such as the mean absolute error (MAE), which is the mean value of individual prediction errors over all instances, root-mean-square error (RMSE), the standard deviation of residuals (prediction errors), and the coefficient of determination (*R*^2^) that measures how well a model predicts an outcome. Classification QSAR models and BNs were validated via multiclass classification metrics^81^ such as balanced accuracy (overall measure of correctly predicted instances with classes having the same weight), precision (true negative rate or specificity), recall (true positive rate or sensitivity), F1 score (weighted average of precision and recall), and Mathews correlation coefficient (MCC), a metric that accounts all confusion matrix categories. In the case of multiclass outcome, those metrics are calculated per class, for example, the metrics for the toxic class consider toxic as true and the union of the remaining classes as false.

## 3 Results

### 3.1 Data Merging and Pre-processing

Table 1 shows the information related to the nanodescriptors and toxicological data. System-independent variables contain information derived by XPS, TEM, and XRD analysis. System-dependent variables contain DLS measurements in two different times. The toxicological data contain information related to (1) in vitro characteristics such as the cell line exposed, the cell type, cell origin, and cell number; (2) exposure conditions such as the exposure dose and duration; and (3) output-related information such as the assay for the cellular viability determination. The FAIR dataset is enriched and annotated with information of the origin of the data, the protocols, and instrumentation and can be found in the Supporting Information and in the open repository Zenodo^k^.

**Table 1:** FAIR Data Gathered Related to Intrinsic Hazard Properties of the Safety Dimension

nanodescriptors	category	protocol	input nanodescriptors	feature type	Unit	missing value imputation
system-independent intrinsic pchem properties. OECD guiding principles: ENV/JM/MONO(2019)	surface properties. Elemental and chemical composition	XPS	Na 1s_Atomic concentration	numerical	%	none
			O 1s_Atomic concentration		%	none
			Ag 3d_Atomic concentration		%	none
			C 1s_Atomic concentration		%	none
			N 1s_Atomic concentration		%	none
		none	coating	categorical		none
	particle size properties	TEM (ISO/DIS 19749)	core size^a^	numerical	nm	none
			spherical surface area^b^		nm^2^	19% → yes
	crystallographic properties^c^	XRD (STAS SR 13203-1994)	crystallinity		%	none
			average crystallite sizes		nm	none
system-dependent extrinsic pchem properties	particle size properties	DLS (ISO 22412:2017)	hydrodynamic size (*Z*-average) *t*_0_		nm	47% → yes
			hydrodynamic size (*Z*-average) *t*_24_		nm	47% → yes
	particle size quality/heterogeneity measurements		polydispersity index *t*_0_			47% → yes
			polydispersity index *t*_24_			47% → yes
biological attributes	in vitro characteristics (human-derived cell lines)	none	organ	categorical		none
			cell line (code)			none
			cell type			none
			multiwell			none
	exposure conditions		exposure dose	numerical	μg/mL (ppm)	none
			exposure duration		h	none
	output related features	Alamar blue, WST-1, MTT [ISO 10993-5:2009]	assay	categorical		none
	output to be predicted		cellular viability	numerical	%	none

a Average cumulative size of 50, 100, and 400 kX magnifications.

b Assumption that the particle is perfectly spherical in shape (*A* = 4*Π*r̂2).

c All the samples in the dataset have a cubic crystal structure, and the amorphous phase is at the crystalline state.

### 3.2 Data Analysis

**Figure 3:**
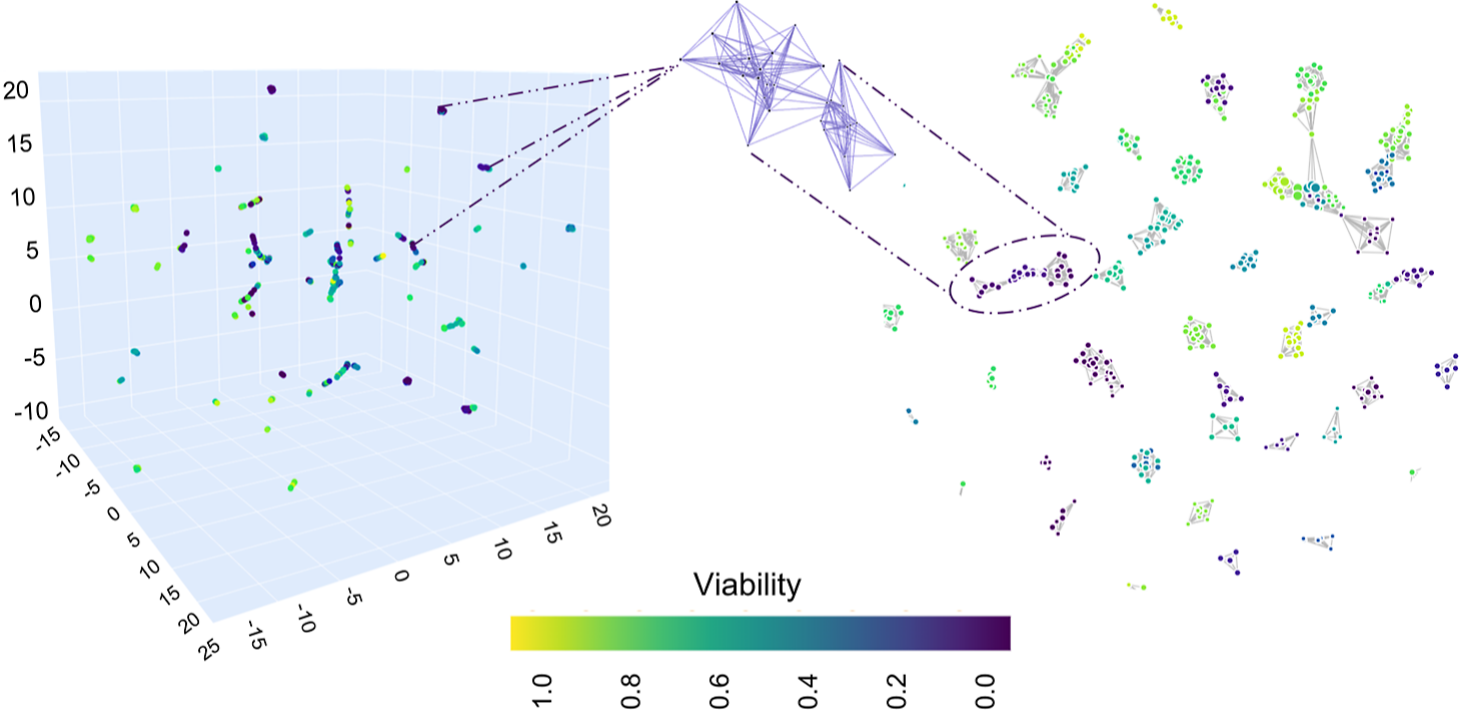
UMAP for dimension reduction (left) and topological network representing the dataset (right). The axes coordinates of UMAP are dimensionless representing an Euclidean space with points distributed so that the low dimensional representation has a similar topological structure to the original data. 3D visualizations of the dataset with respect to viability are colored by a scaled order of viability.

In Figure 3, UMAP and Mapper topological network projecting data into lower dimensional spaces demonstrating the structure of the data. Even with missing value imputations, the experimental data are quite sparse with relatively low variance, with the local dimension varying across the data and the dataset uniformly distributed on the Riemannian manifold (Figure 3, left). This was expected since the dataset contains (i) triplicates of toxicological experiments (rows with identical inputs but different outcomes) and (ii) rows where the only feature varying is the exposure dose or the assay. UMAP places related experiments near to one another with the color differentiating the 3D experimental space based on cellular viability-scaled values. The gaps between the points signify the experiments that could have been hypothetically performed.

Data are projected into a two low-dimensional simplicial complex with the Mapper algorithm inclosing clusters varying by color and size containing cubes (Figure 3, right). The color indicates the value of the function at a representative point (cell viability), and the size indicates the number of dots in the set (experiments), providing information about the nature of the output.^68^ Each dot belongs to a rule/criteria (cube) and finding the input’s association of the cubes within the cluster provides the output.

**Figure 4:**
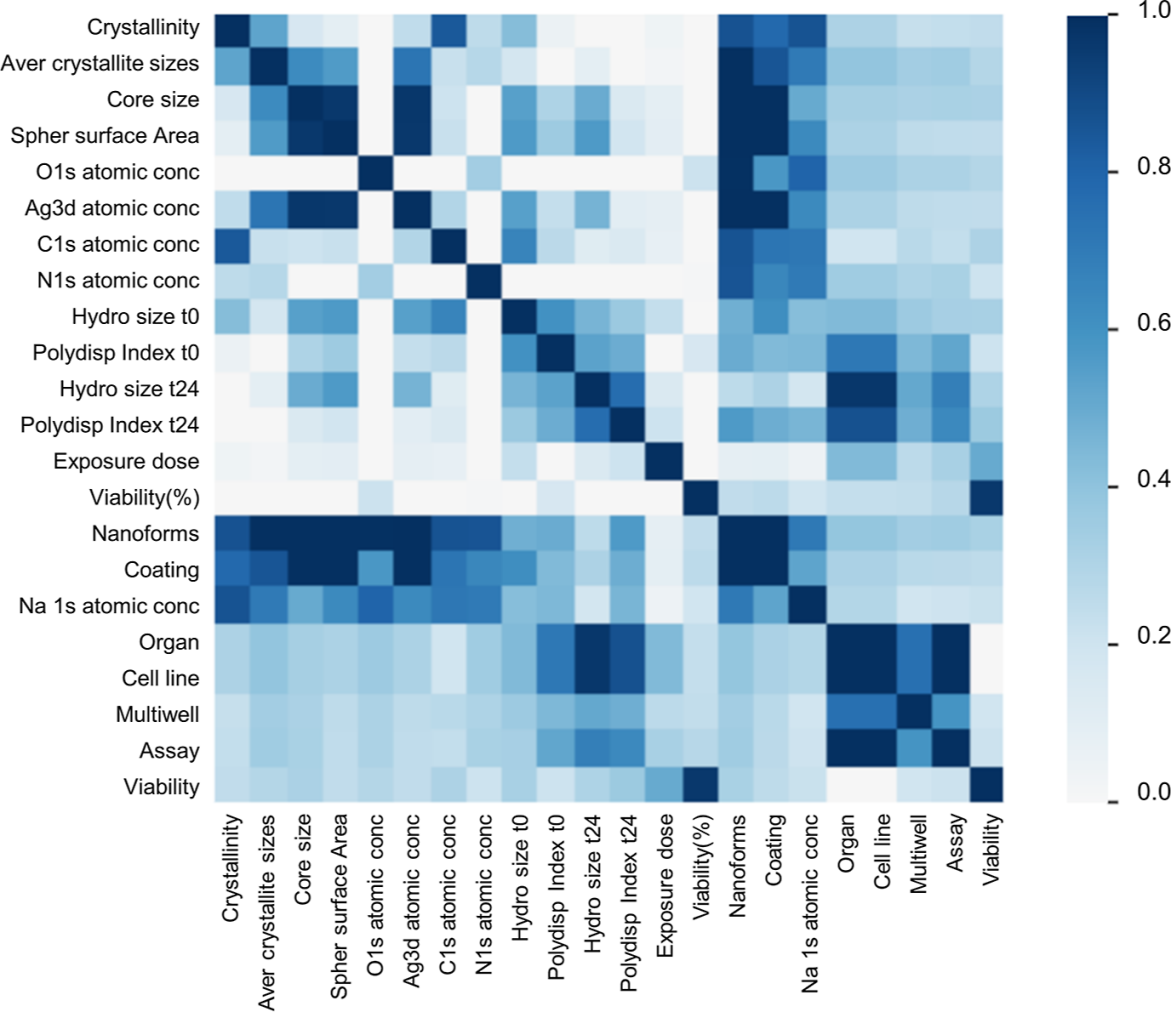
Inter-relationship correlations of input variables and between the inputs and output.

The pairwise correlation among the features based on Spearman’s ρ for the numeric features and based on Cramer’s V for the categorial relationships is shown in Figure 4.

The output (viability expressed either as % or multiclass) shows no correlation with input features. The NFs (ERM identifiers) which are utilized during missing value imputation with the lightgbm algorithm shows high correlation with nearly half of the features in the dataset. Thus, including it during the imputation helped the algorithm to efficiently allocate missing values. Crystallinity is correlated with the C 1s atomic concentration, while Ag 3d atomic concentration is correlated with the core size, coating, and surface area of NFs. The average crystallite sizes are correlated with the coating along with other features such as the C 1s atomic concentration. This information is useful for the reasoning construction of the BN structure since some correlation among features is required. Negative linear correlation is shown among O 1s with Ag 3d and C 1s atomic concentrations (see Supporting Information Figure S1: Pearson *r* correlation). The cell line is highly correlated with the organ, multiwell, assay, and the hydrodynamic size at *t*_24_. Thus, we kept only the cell line as a final feature to be used in the modeling part since it encapsulates the information regarding the organ. Such input features would be valuable in case of diverse targeted organs to reveal any target-specific toxicities. Assay is kept which includes information of the multiwell used. Regarding the correlation of the above-mentioned features with hydrodynamic size at *t*_24_, correlation does not signify causation, and this information is not deemed redundant in our case. In BNs, determining the conditional dependencies among the features goes beyond the correlation concept revealing the causal effect probabilities among the features (Pearl’s ladder of causation).^82^

Table 2 shows the final modeling features along with their skewness and the transformed bins for the BN training, which also represent the applicability domain of the QSAR model. Skewness quantifies distribution asymmetry, and values between −2 and +2 are acceptable to demonstrate a normal univariate distribution.^83^ All features show good skewness except hydrodynamic size at *t*_24._ However, the feature is included since it contributes greatly to the information gain analysis of the dataset (see Supporting Information Table S1: attribute selection). All the experiments refer to a 24 h acute in vitro toxicological screening, thus from the exposure conditions, only the exposure dose was considered. Na 1s and N 1s atomic concentrations were not considered for the modeling due to redundant zero vales and the fact that those features are related to the synthesis process and precursors utilized and have no causal effect to hazard effects.

**Table 2:** Features in the Final Dataset for Modeling Purposes^a^

input features	metric	skewness	bins [for the BN structure training]
O 1s_Atomic	%	0.78	“low”: [14.16 → 14.54], “medium”: [14.54 → 34.02], “high”: ≥34.02
Ag 3d_Atomic	%	–0.21	“low”: [0.08 → 0.19], “medium”: [0.19 → 15.53], “high”: ≥15.53
C 1s_Atomic	%	–0.19	“low”: [54.23 → 60.61], “medium”: [60.61→ 61.83], “high”: ≥61.83
core size	nm	0.60	“low”: [7.0 → 17.8], “medium”: [17.8 → 20.00], “high”: ≥20.00
spherical surface area	N m^2^	1.03	“low”: [3981.53 → 3981.59], “medium”: [3981.59→ 5023.55], “high”: ≥5023.55
crystallinity	%	1.61	“low”: [22.9 → 60.0], “medium”: [60.0 → 61.0], “high”: ≥61.0
av crystallite sizes	nm	0.35	“low”: [46.0 → 98.0], “medium”: [98.0 → 117.0], “high”: ≥117.0
coating			HEC, PVP, CUR, none (one hot encoded)
hydrodynamic size *t*_0_	nm	1.79	“low”: [55.91 → 209.97], “medium”: [209.97 → 363.31], “high”: ≥363.31
hydrodynamic size *t*_24_	nm	2.67	“low”: [63.74 → 149.34], “medium”: [149.34→ 266.90], “high”: ≥266.90
pol index *t*_0_		–0.72	“low”: [0.18 → 0.47], “medium”: [0.47 → 0.59], “high”: ≥0.59
pol index *t*_24_		0.31	“low”: [0.04 → 0.28], “medium”: [0.28 → 0.49], “high”: ≥0.49
cell line			A549, HCT-116 (one hot encoded)
exposure dose	ppm	0.32	“low”: [0.0 → 20.0], “medium”: [20.0→ 58.35], “high”: ≥58.35
assay			WST-1, MTT, Alamar blue (one hot encoded)
output feature	metric	skewness	bins [for the BNs structure training]
cellular viability	%	–0.28	very toxic [0 → 30.0%], toxic [30.0 → 70.0%], safe >70%

a 900 rows transformed into the final dataset of 1682 rows through SMOTE implementation for the classification modeling and BNs. The bins also demonstrate the applicability domain of the QSAR model based on BN algorithm in which the model makes predictions with a given reliability.

### 3.3 QSAR Development and Validation

#### 3.3.1 ML Exploration

QSAR models trained either as regression or classification ML tasks are able to predict cellular viability with satisfactory results. Table 3 shows the top three regressor and classifier algorithm’s external performance metrics. Random forest regressor (rf) slightly outperforms the other regressors achieving *R*^2^ = 0.7, MAE = 12.77, and RMSE = 19.55. Extra trees classifier (et) faintly outperforms rf, reaching a balanced accuracy of 85%, a F1 score (a harmonized metric including precision and recall) of ≈85%, and a MCC of 77%. Additional algorithms with their internal 10-fold cross-validation, hyperparameterization, and external performance metrics can be found in the Supporting Information (Tables S2–S5: additional algorithms’ validation metrics).

**Table 3:** Performance Metrics (External Validation/Predictivity with 20% of the Dataset) for the Top Three Regressors and Classifiers

			performance metrics		
task		models	MAE	RMSE	*R*^2^
regression	rfr	random forest regressor	12.77	19.55	0.70
	lightgbm	light gradient boosting machine	12.91	19.71	0.70
	gbr	gradient boosting regressor	14.17	20.18	0.68
			ACC	F1	MCC
classification	et	extra trees classifier	0.85	0.85	0.77
	rf	random forest classifier	0.84	0.84	0.77
	lightgbm	light gradient boosting machine	0.84	0.83	0.76

#### 3.3.2 BNs and Rules Extraction

**Figure 5:**
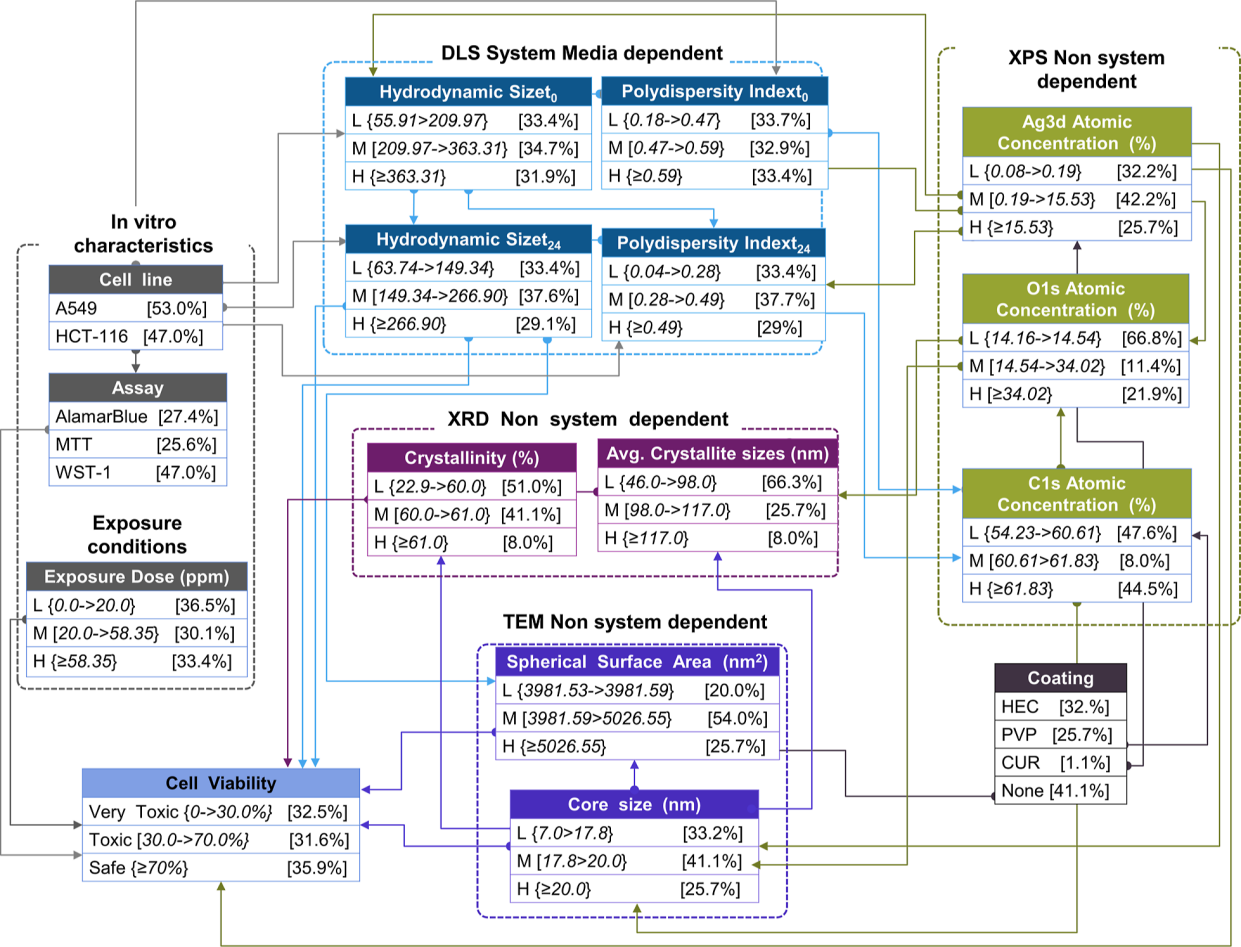
Graphical structure of the constrained BN representing the variables (input features and toxicological attributes) along with the conditional probabilities. Arcs represent the conditional dependencies between the features. The different color represents the categories of the input features containing system-dependent and system-independent pchem properties measured with different protocols, biological attributes (in vitro characteristics), and exposure conditions.

For the development of the constrained reasoned structured network, expert judgment was applied to conditional dependencies. Some alterations of arcs include polydispersity index *t*_24_ and hydrodynamic size *t*_0_, where *t*_24_ features were parents to exposure dose in the unconstrained structure (see Supporting Information Figure S2: unconstrained BN structure); however, such a dependency is not realistic; that is, the external exposure dose cannot be determined by the hydrodynamic size; however, the exposure dose did fed the cell viability node, which is kept in the constrained structure, eliminating the other relationships. The exposure dose was forced to act as an independent global parameter^84^ (see Figure 5, node: exposure dose, color gray). The same reasoning was applied for the assay, which affects the output to be predicted. Coating is fed by the core size in the unconstrained structure, but knowing the coating has no effect on predicting the core size; instead, the coating feature determines the surface area and the Ag 3d atomic concentration in the case of uncoated AgNFs. Polydispersity index at *t*_0_ appeared at the end of the structure with hydrodynamic size at *t*_0_ and the cell line feeding it. Thus, one constrain included in the structure was that the output should be the only feature receiving prior knowledge at the terminate point. Core size fed many nodes in the unconstrained structure, and the pattern was kept in the constrain as well. Features measured at *t*_0_ should act like parents to features at *t*_24_ and not vice versa (see Figure 5, system media dependent, color blue). The constrained structure follows a reasoning pathway of scale information starting from a higher level such as coating to → structure level → to atomic system-independent properties, while the system-dependent features are fed by medium information encapsulated within the cell line. With the above manipulations of the conditional dependencies based on expert judgment, the constrained model displayed slightly higher predictive capacity for some classes, but most importantly, the structure has reasoning, in order for the rules to make sense.

Exploring the Weka software for automated BN construction (estimator: simple, *a* = 0.5, search algorithm: local hill climber, six parents limit configuration), the unconstrained structure also demonstrated the exposure dose, hydrodynamic size at *t*_24_, core size, and assay being connected to the outcome, reinforcing the reasoning on some arcs (see Supporting Information Figure S3: unconstrained Bayesian structure derived from WEKA software).

**Figure 6:**
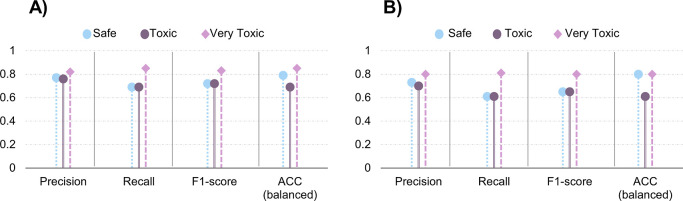
External validation metrics containing precision (PREC), recall (REC), and F1 score and balanced accuracy (ACC) per class label of the constrained structure (A) and the unconstrained structure (B).

Figure 6 demonstrates the external validation results of the QSAR BN-based model tested with the 20% test set. The metrics are quite similar for both structures. However, we demonstrate that even with constrains, the predictability of the BNs is slightly increased (MCC for unconstrained: 62% vs constrained 67%, data not shown in figure since MCC considers all classes into a single metric).

The focus on the validation was to surpass the performance for the very toxic instances from the structure learned with no reasoning. The constrain model has higher performance metrics for the very toxic instances. This is significant especially in the case of the high recall (REC: 85% constrained vs 81% unconstrained), meaning a false negative instance (safe or toxic) rarely gets predicted instead of very toxic. The BN performed better also for safe instances (PREC: 77% constrained vs 73% unconstrained). Regarding the toxic instances, the constrained BN scores 69% ACC (vs 61% unconstrained).

##### 3.3.2.1 Rules Extraction

The extraction of the interpretable rules related to the quantitative intrinsic hazard properties of AgNFs was filtered down to the cases where the hazard class was present and with the highest CF as an example. For the theoretical scenarios where the input ranges fall outside those given rules (or the ones provided in the Supporting Information, Section 4: extra rules), Bayesian inference (or the regressors/classifiers) can be used to determine the hazard class with a given CF. The higher the CF, the higher the posterior probability of that statement/rule to be true. Infinite confidence probabilities, an instance that occurs due to a divide-by-zero runtime exception when comparing the likelihood of events with no counterexamples, were discarded.

The following rules are mentioned based on the BN structure’s CPTs solely as examples, where L denotes low, M medium, H high values in the representative bins (see Table 2), and ^ the logical symbol for *and*:

(1) Quantitative intrinsic hazard criteria for lung cells (under MTT assay)

IF (crystallinity) = M(60 → 61)^core size = M(18 → 20)^spherical surface area = M(3982 → 5026)^Ag 3d at. % = M(0.2 → 15)^hydrodynamic size *t*_24_ = L(64 → 149) THEN AgNFs are toxic if tested under low (0.0 → 58) dose with an average 0.82 probability.

IF (crystallinity) = H(>61)^core size = L(7 → 18)^spherical surface area = L(3981.53 → 3981.59)^Ag 3d at. % = L(0.08 → 0.2)^hydrodynamic size *t*_24_ = L(64 → 149) THEN AgNFs are safe if tested under low (0.0 → 58) dose with an average 0.81 probability.

(2) Quantitative intrinsic hazard criteria for lung cells (under Alamar blue assay)

IF (crystallinity) = L(23 → 60)^core size = L(7 → 18)^spherical surface area = M(3982 → 5026)^Ag 3d at. % = L(0.08 → 0.2)^hydrodynamic size *t*_24_ = M(149 → 267) OR.

IF (crystallinity) = H(>61)^core size = L(7 → 18)^spherical surface area = L(3981.53 → 3981.59)^Ag 3d at. % = L(0.08 → 0.2)^hydrodynamic size *t*_24_ = L(64 → 149) THEN AgNFs are very toxic if tested under low dose (0 → 20) or medium dose (20 → 58), respectively, with a 0.87 probability (CF = 6.7).

(3) Quantitative intrinsic hazard criteria for intestinal cells (under WST-1 assay)

IF (crystallinity) = L(23 → 60)^core size = L(7 → 18)^spherical surface area = M(3982 → 5026)^Ag 3d at. % = L(0.08 → 0.2)^hydrodynamic size *t*_24_ = M(149 → 267) THEN AgNFs are safe if tested under any exposure range with an average 0.87 probability (CF = 35).

The rules mentioned are only a sub-part of all the rules and serve as an technical extract example of the model that can be used as a formula, ad hoc. The structure of the BN contains the decision structure and the rules within—a total of rules extracted from this case is ∼150.

## 4 Discussion

### 4.1 Data

For the moment, a great amount of information produced by H2020 projects is stored online in private servers, locked to external users, making the data re-usability unfeasible, hindering progress and data integration, especially for modeling purposes.^85^ Commmission^4^ remarks that research and innovation are needed in open platforms to ensure access and data integration from different databases enabling exchanges between different stakeholders in line with data governance acts, meaning an overarching level.^1^ Metadata capturing is not frequently promoted in regular academic practice, despite its importance. This is due to a lack of data management training. This FAIR challenge requires the active involvement, participation, and collaboration of participants with different expertise. In this work, we used data captured by the data shepherd,^8^ which demonstrates that this role is essential in a project where data are generated, modeled, and used.^85^ The role of the shepherd is to capture data, protocols, and instrumentations and to help with data reporting, merging, and harmonization. The most important part of this role is the implementation of the FAIRification process with multiple stakeholders who are unaccustomed with the notion of FAIRification process.^8−10^ It is outside of the scope of this manuscript to provide details regarding the FAIR initiatives and the efforts in place in the EU. The reader can refer to the following footnotes^l^^,^^m^ to get an appreciation of the current initiatives regarding FAIR data.

The size of the dataset used in this study is not remarkable (in comparison to common experimental computer science fields), but in comparison to the dataset sizes used in the nanocomputational domain literature, the data size is sufficiently large.^16^ To tackle this sparsity, the approach in this work is twofold, the data is augmented by a standard method to oversample sparse data with SMOTE leading to 1682 data points and by applying BN ML algorithm, which is a robust learning paradigm in the sparse data regime. In addition, the interoperability of data is high due to the annotation with ontological identifiers from eNanoMapper and with ERM identifiers which ensure that internal project documentation can later be linked to released data for specific NFs.^35^ The dataset contains harmonized features derived from different laboratories; for example, the system-dependent properties reported in the toxicological datasets were similar across the two partners, greatly facilitating the merging. By capturing the measurements at two time points, we were able to account for variations that might occur to the size when dispersed and once when in contact with cellular medium.^47^ By incorporating the polydispersity index, we increases the quality of the measurements and consequently the modeling.^45^ The data is targeting cellular viability, which is the majority of the re-usable data that exist in the literature and databases,^27,85^ implying a potentiality of further merging. Also, exposure conditions and in vitro characteristics are commonly considered as input features.^59^ The exposure aspects are not part of step 1—hazard assessment of the SSbD framework.^3^ In this work, we go a step further considering, besides the system and non-system-dependent properties, also the in vitro characteristics and exposure conditions to better represent how those properties are altered depending on the dose and the cellular target. We argue that inherently safer NFs should be cell line (organ)-target specific; that is, NFs safe for skin cells could be harmful for lung cells. Taking into account in vitro features for the hazard criteria, we capture the dynamic and complex nature of NFs when surrounded by a biological environment.^43^ In addition, from a toxicological point a view, dose should be considered at each dimension. Including exposure conditions increase the performance as this information is always reported in in vitro studies but could also reduce the biological accuracy by grouping this information in a node of exposure features not readily comparable; for example, exposure doses for different tissues cannot be grouped. However, the nodes were included as exposure criterion, which is a crucial variable in the hazard notion.

### 4.2 Data Pre-processing

Commission^4^ mentions the need of improved methods to address missing data such as ML-based methods. In this study, an iterative sequential imputation process was executed via regression with the lightgbm algorithm. Other techniques have been proposed such as a hybrid missing data imputation method incorporating records similarity using the global correlation structure by using *k*-nearest neighbors and iterative imputation algorithms^86^ or by merits integration of decision trees and fuzzy clustering into an iterative learning approach.^87^

A quantile-based discretization function was performed in this study to discretize features into bins. For this step, alternative methods have been proposed, for example,^88^ introduced a dynamic programming search strategy and a Bayesian score for the evaluation and the discretization of variables.

UMAP places related experiments (each row of the dataset) near to one another. Such an approach could hypothetically be helpful to identify the experimentations that should be prioritized during a project, in a data gap filling manner, supporting the application of QSAR modeling. Since the axes in UMAP are non-dimensional, input features could be used to predict *x*, *y*, *z* values. On a second step, a SHapley Additive exPlanations (SHAP) analysis could reveal the most important features determining the space and were experimentations should focus.^89^ In addition, the dimensionality reduction algorithms could be more interpretable only for some cases due to complexity.^19^ However, this field is under research, with hyperparameter choice appearing to play an important role.

### 4.3 NAMs

QSAR models based on random forest (rf) and extra tress (et) algorithms showed good validation metrics in our study. Throughout the literature, rf has been shown to surpass other algorithms.^90−92^ Et algorithm generates a large number of unpruned decision trees from the dataset and then combines the predictions. Et similarly to rf randomly samples the features at each split point. However, et splits the nodes by selecting cut points randomly, in comparison to rf, and fits each decision tree to the entire training dataset whose structures are independent of the output values.^93^

The theoretical framework from ref (3) and the recent report by ref (4) both mention NAM approaches as helpful tools in the implementation and validation of the SSbD approach, without providing instructions. This study is a contribution of an iterative consolidation of modeling and experimental domain expertise. We demonstrate how experimentalists in conductions with modelers can act in a complementary manner, accelerating the progress in the nanosafety domain. Bringing the gaps between the three fields (toxicology, material designers, and modelers) demanded strong communication, interaction, while transferring experimental domain knowledge, adopting a multidisciplinary approach.^94^ In this work, we demonstrated in a detailed manner how QSAR tools based on BNs coupled with expert judgment can be used for the definition and extraction of quantitative intrinsic hazard criteria. The same approach can be used in datasets targeting different outputs.

The modeling approach is unique in some points: (i) the BN model is crafted by expert reasoning integrating system-dependent and -independent nanodescriptors in combination with in vitro experimental conditions derived from a FAIR process to predict a biological effect, (ii) the data refer to NFs that have the same chemical identity but a unique fingerprint that allows a NF-dependent differentiation among the same substance, (iii) the interpretable rules can guide material developers into synthesizing (re-synthesizing) inherently safer NFs, and (iv) the models (BN, regressors, and classifiers) can enable the fast and cost-efficient in silico toxicological screening of previously synthesized NFs and hypothetical scenarios of yet-to be synthesized NFs. It is worth noting that the methodology strongly improves given variables that material designers have the most control over modifying in the laboratory. For the development of the BN structure and the CPTs, we utilized an open source ML package pomegranate.^76^ Other packages for the implementation of BN are documented.^95^

In the nanosafety, there are no clear understandings of causal relations among nanodescriptors and hazardous attributes, only statistical relevance information. Such relevance is insufficient to fully capture causal relations. This means that any explanation proven wrong may have to be prohibited within the structure.^78^ The BNs can perform an incremental learning, meaning, as more data become available in the nanosafety domain, the existing structure can remain the same, or updated to novel modifications of parameters (inclusion of additional nanodescriptors or hazard endpoints), and even a new structure, to fit the new data.^22^ The BN can also perform with multiple outcomes, rendering it an optimum solution in the case of multiple hazard criteria^96^ while also providing a robust learning paradigm in the sparse data regime.^97^ The extraction of the rules from the CPTs is performed with the aim to extract quantitative intrinsic hazard design criteria that can be used in SSbD paradigms. The interpretable rules can act in a hierarchic manner, meaning that the last descriptors have to be measured only if the previous IF statements are met. Identifying quantitative criteria to address the SSbD multicriteria decision problem is one of the most significant goals where collective robust efforts are currently placed. The rules are followed with CFs, which is the likelihood ratio for and against an outcome when presented with evidence, as a means of expressing domain knowledge and creating expert systems that can take into account quantitative uncertainties. The quantifiable CFs for each rule deliver a convenient system to manage uncertainties in a criteria-based framework. As a result, such a rule-based system will have practical ways to elicit expert knowledge and clearly communicate the reasoning process. This methodology proposed entails a flexible, nuanced, and promising approach applicable at each SSbD dimension with a goal to extract a set of quantitative criteria in a data-driven manner.

## 5 Conclusions

Collaborative efforts are required among data shepherds, experimentalists, experts, and modelers to merge information in an iterative manner that can reveal valuable information for each SSbD dimension. BNs are promising probabilistic ML tools helpful to (i) derive interpretable rules from FAIR data, (ii) capable and flexible in updating their conditional dependencies from new data while (iii) allowing the quantification of the uncertainties. In addition, they present graphical structures developed from expert reasoning in combination with automated inference. In this work, utilizing system (i.e., hydrodynamic size and polydispersity index) and non-system (i.e., elemental composition and core size)-dependent nanodescriptors in combination with biological in vitro attributes and experimental conditions, we demonstrate how such a methodology can be used for extracting quantitative intrinsic hazard criteria for silver NFs, synthesized with the intend of antimicrobial/antiviral functional textiles and antimicrobial creams or lotions (cosmetics) applications, which can guide materials designers toward intrinsically safer materials while saving time, effort, and money for the toxicologists.

## Supporting Information Available

FAIR dataset used for the modeling can be found in the supplementary file and in open repository Zenodo (https://zenodo.org/record/7335039#.Y3emqnbMIQ8) (XLSX)
Additional data analysis, QSAR development, validation metrics of algorithms, and additional BN rules extracted (PDF)

## Footnotes

a https://zenodo.org/record/4652587#.Y1eVGnZBwQ8.

b The data shepherd not only oversees the data management, handling, and quality control processes but also communicates in a simple language with all parties. Data shepherds combine experimental, computational, and technical background and lead the data quality control and FAIRness evaluation facilitating the data curation.^6^

c https://webcast.ec.europa.eu/3rd-meeting-of-the-high-level-roundtable-on-the-chemicals-strategy-for-sustainability [Accessed November 2022].

d https://www.youtube.com/watch?v=R3_QEZIShf0&t=1578s [Accessed November 2022].

e https://www.asina-project.eu/asina-1st-stakeholder-workshop-bioceramics-2022/[Accessed November 2022].

f https://www.h2020sunshine.eu/events/nanosafety-training-school-venice-2022 [Accessed November 2022].

g https://eur-lex.europa.eu/legal-content/EN/TXT/?uri=COM%3A2020%3A667%3AFIN [Accessed November 2022].

h Patent number granted in USA: US10525432B2.

i https://www.iso.org/standard/65410.html [Accessed November 2022].

j https://one.oecd.org/document/ENV/JM/MONO(2016)7/en/pdf [Accessed November 2022].

k https://zenodo.org/record/7335039#.Y3emqnbMIQ8.

l https://www.go-fair.org/implementation-networks/overview/advancednano/[Accessed November 2022].

m https://worldfair-project.eu/[Accessed November 2022].
