# Supplementary material for: Data-Driven Quantitative Intrinsic Hazard Criteria for Nanoproduct Development in a Safe-by-Design Paradigm: A Case Study of Silver Nanoforms
Source: ACS Appl Nano Mater. 2023 Feb 16;6(5):3948–62. doi: 10.1021/acsanm.3c00173 (PMC10012170; doi:10.1021/acsanm.3c00173)
Supplement: Supplementary file 2 — an3c00173_si_002.pdf [file an3c00173_si_002.pdf]

Data-Driven Quantitative Intrinsic Hazard Criteria for Nanoproduct Development in a Safe-by-Design Paradigm: A Case Study of Silver Nanoforms.

Irini Furxhi[1,2], Rossella Bengalli[3], Giulia Motta[3], Paride Mantecca[3], Ozge Kose[4], Marie Carriere[4] Ehtsham Ul Haq[5], Charlie O'Mahony[5], Magda Blosi[6], Davide Gardini[6] and Anna Costa[6]*

[1]Transgero Ltd, Limerick, V42V384, Ireland.

[2]Dept. of Accounting and Finance, Kemmy Business School, University of Limerick, V94T9PX, Ireland.

[3] University of Milano-Bicocca, Dept. of Earth and Environmental Sciences, Piazza della Scienza 1, 20126 Milano , Italy

[4]Univ. Grenoble Alpes, CEA, CNRS, Grenoble INP, IRIG, SYMMES, 38000 Grenoble, France

[5]Department of Physics, and Bernal Institute, University of Limerick, Limerick V94T9PX, Ireland

[6]Istituto di Scienza e Tecnologia dei Materiali Ceramici (CNR-ISTEC), Via Granarolo, 64, 48018 Faenza RA, Italy

*Correspondence: irini.furxhi@transgero.eu, irini.furxhi@ul.ie. Tel: +353 85 106 9771

Experimental Section

1 Data Preprocessing

1.1 Attribute selection

Information gain analysis of the training dataset (80%) based on average impurity decrease (and number of nodes using that attribute). Search Method: Attribute ranking. Attribute Evaluator (supervised, Class (nominal): 16 viability): Information Gain Ranking Filter. Selected attributes: 9,11,10,12,13,6,3,7,2,4,8,5,1,15,14.

Table S1- *Attribute Selection.*

**Information Gain**		**Ranked attributes**
**0.656963**	9	Hydro_size_t0_nm
**0.647354**	11	Hydro_size_t24_nm
**0.582135**	10	PI_t0
**0.573924**	12	PI_t24
**0.565378**	13	Exposure dose_PPM
**0.153672**	6	O 1s_Concent_%
**0.153672**	3	Aver_crystallite_sizes_nm
**0.153672**	7	Ag 3d_Concent_%
**0.153672**	2	Crystallinity_%
**0.150748**	4	Core size
**0.146015**	8	C 1s_Concent_%
**0.107264**	5	Spherical_surface_Area_nm^2 _TEM
**0.098697**	1	Coating
**0.069231**	15	Assay
**0.00051**	14	Cell_line

1.2 Correlation Analysis

The Pearson's correlation coefficient (r) is a measure of linear correlation between two variables. It's value lies between -1 and +1, -1 indicating total negative linear correlation, 0 indicating no linear correlation and 1 indicating total positive linear correlation. Furthermore, r is invariant under separate changes in location and scale of the two variables, implying that for a linear function the angle to the x-axis does not affect r.

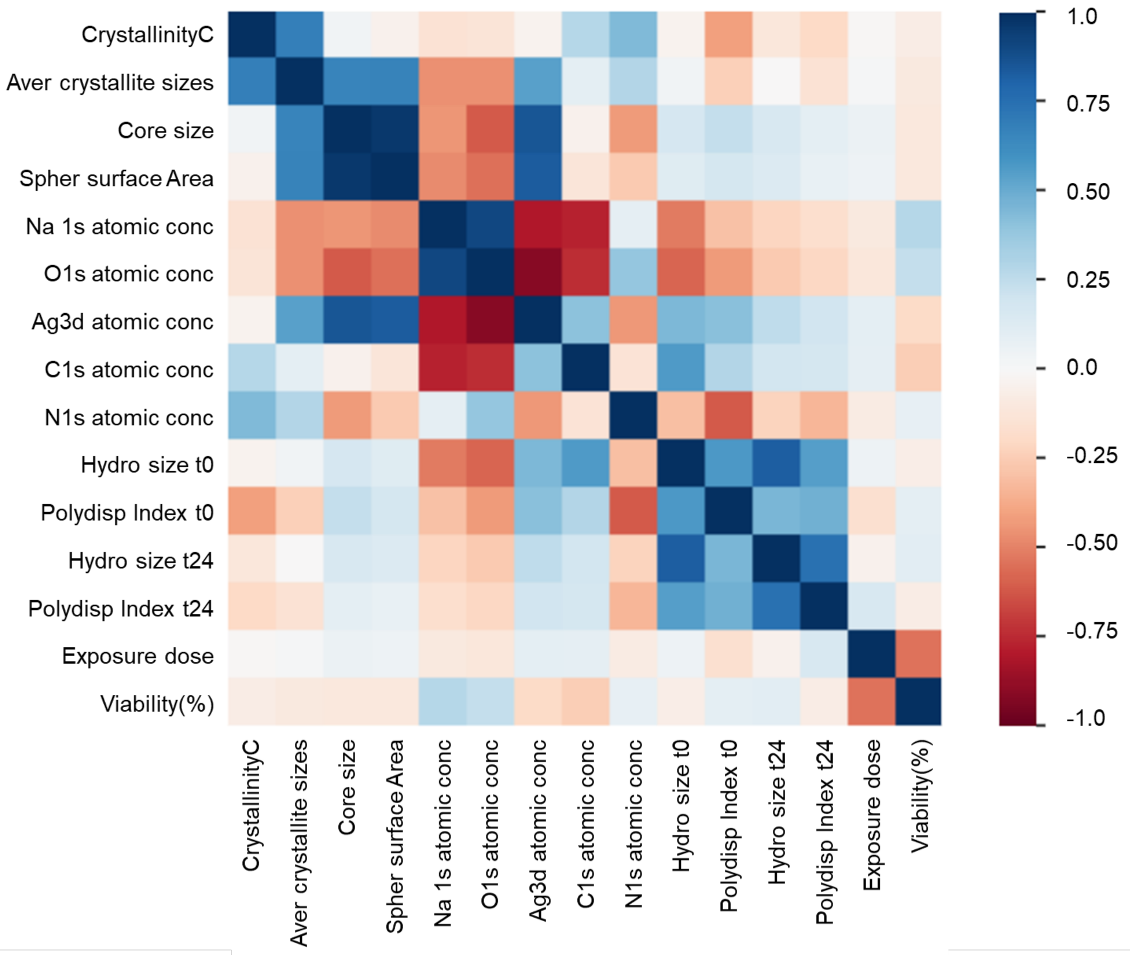

Figure S1- *Pearson r correlation.*

2 QSAR development and Validation

2.1 Regression algorithms

QSAR models trained either as regression or classification ML tasks, are able to predict cellular viability with satisfactory results. Additional algorithms with their internal 10-fold cross validation, hyper parameterization and external performance metrics.

Table S2- *Additional regression algorithms external predictivity performances metrics. Regression algorithms are derived from PyCaret library.*

	Model	MAE	MSE	RMSE	R2	RMSLE	MAPE	TT (Sec)
**rf**	Random Forest Regressor	1.277600e+01	3.874638e+02	1.955080e+01	7.053000e-01	0.8154	3.369500e+00	1.657
**lightgbm**	Light Gradient Boosting Machine	1.291420e+01	3.952788e+02	1.971850e+01	7.000000e-01	0.8147	3.268300e+00	0.212
**gbr**	Gradient Boosting Regressor	1.417130e+01	4.120250e+02	2.018230e+01	6.875000e-01	0.8601	3.445100e+00	0.406
**et**	Extra Trees Regressor	1.265830e+01	4.220538e+02	2.035510e+01	6.784000e-01	0.8292	3.334900e+00	1.123
**dt**	Decision Tree Regressor	1.352500e+01	5.156505e+02	2.256230e+01	6.064000e-01	0.8843	3.324700e+00	0.046
**ada**	AdaBoost Regressor	1.757810e+01	5.262554e+02	2.292110e+01	6.010000e-01	0.9418	3.740200e+00	0.073
**ridge**	Ridge Regression	1.752050e+01	5.373334e+02	2.310810e+01	5.924000e-01	1.0021	3.595300e+00	0.053
**br**	Bayesian Ridge	1.767000e+01	5.426961e+02	2.322050e+01	5.885000e-01	1.0093	3.674800e+00	0.046
**lasso**	Lasso Regression	1.846020e+01	5.711001e+02	2.382630e+01	5.681000e-01	1.0228	3.890700e+00	0.075
**knn**	K Neighbors Regressor	1.627680e+01	5.909606e+02	2.424100e+01	5.548000e-01	0.9469	4.405700e+00	0.065
**en**	Elastic Net	1.950880e+01	6.404212e+02	2.521680e+01	5.167000e-01	1.0267	4.475900e+00	0.090
**omp**	Orthogonal Matching Pursuit	2.041900e+01	6.621426e+02	2.568250e+01	4.971000e-01	1.1234	4.110000e+00	0.036
**lr**	Linear Regression	1.915100e+01	6.833052e+02	2.563940e+01	4.862000e-01	1.0358	3.684100e+00	0.030
**huber**	Huber Regressor	2.626660e+01	1.183424e+03	3.429290e+01	1.029000e-01	1.1137	4.961900e+00	0.307
**llar**	Lasso Least Angle Regression	3.208570e+01	1.347116e+03	3.667230e+01	-1.860000e-02	1.3682	7.244700e+00	0.026
**dummy**	Dummy Regressor	3.208570e+01	1.347116e+03	3.667230e+01	-1.860000e-02	1.3682	7.244700e+00	0.005
**par**	Passive Aggressive Regressor	1.410491e+02	4.597176e+04	1.845018e+02	-3.388820e+01	2.0405	2.633870e+01	0.024
**lar**	Least Angle Regression	7.576682e+08	6.058722e+20	8.153330e+09	-4.371112e+17	9.6370	6.391748e+08	0.053

Table S3- *Internal 10-fold validation of lightgbm regression algorithm (left) and hyperparameters tuning (right) results.*

Fold	MAE	MSE	RMSE	R2	RMSLE	MAPE
0	13.0210	362.8833	19.0495	0.6939	0.6374	1.0460
1	11.9836	327.1305	18.0867	0.7648	0.8972	3.2214
2	12.6223	386.5662	19.6613	0.6944	0.8521	3.2695
3	10.8628	231.2104	15.2056	0.8480	0.7137	1.5745
4	10.3974	268.6802	16.3915	0.8230	0.6744	1.3669
5	12.9043	472.9354	21.7471	0.6776	0.9504	3.6544
6	15.2028	610.9565	24.7175	0.5585	1.0391	4.9407
7	13.0543	378.5734	19.4570	0.7176	0.6915	1.4132
8	13.6376	406.4968	20.1618	0.6919	0.7040	2.4023
9	11.5706	355.9808	18.8675	0.7255	0.6983	1.9183
Mean	12.5257	380.1413	19.3345	0.7195	0.7858	2.4807
Std	1.3260	100.4592	2.5133	0.0768	0.1307	1.1909

Fold	MAE	MSE	RMSE	R2	RMSLE	MAPE
0	13.3310	361.6793	19.0179	0.6949	0.6262	1.1164
1	11.8613	307.3331	17.5309	0.7790	0.8991	3.0610
2	12.7953	397.2119	19.9302	0.6860	0.8678	3.1065
3	10.9606	243.4240	15.6021	0.8399	0.7172	1.5383
4	11.0776	283.5458	16.8388	0.8132	0.6833	1.3283
5	12.9984	429.9490	20.7352	0.7069	0.8982	3.3964
6	14.7015	523.7986	22.8866	0.6215	1.0192	4.5988
7	12.5535	337.8803	18.3815	0.7480	0.7090	1.3543
8	14.1569	434.6923	20.8493	0.6705	0.7560	2.5743
9	11.3882	328.3799	18.1213	0.7468	0.6792	1.8345
Mean	12.5824	364.7894	18.9894	0.7307	0.7855	2.3909
Std	1.2079	78.8404	2.0477	0.0641	0.1206	1.0838

2.2 Classification algorithms

Table S4- *Additional classification algorithms external predictivity performances metrics. Classification algorithms are derived from PyCaret library.*

	Model	Accuracy	AUC	Recall	Prec.	F1	Kappa	MCC	TT (Sec)
et	Extra Trees Classifier	0.8530	0.9352	0.8503	0.8560	0.8527	0.7781	0.7798	1.120
rf	Random Forest Classifier	0.8463	0.9489	0.8428	0.8503	0.8456	0.7678	0.7701	1.552
lightgbm	Light Gradient Boosting Machine	0.8403	0.9478	0.8370	0.8439	0.8397	0.7586	0.7608	0.286
gbc	Gradient Boosting Classifier	0.8267	0.9475	0.8221	0.8290	0.8253	0.7381	0.7403	0.749
dt	Decision Tree Classifier	0.8199	0.8969	0.8157	0.8257	0.8195	0.7278	0.7307	0.020
knn	K Neighbors Classifier	0.7757	0.9114	0.7728	0.7837	0.7761	0.6610	0.6637	0.057
ada	Ada Boost Classifier	0.7367	0.8333	0.7359	0.7440	0.7377	0.6041	0.6061	0.183
lda	Linear Discriminant Analysis	0.6814	0.8585	0.6728	0.6813	0.6763	0.5189	0.5228	0.035
ridge	Ridge Classifier	0.6602	0.0000	0.6457	0.6546	0.6377	0.4825	0.4961	0.030
lr	Logistic Regression	0.6380	0.8127	0.6294	0.6403	0.6296	0.4512	0.4580	0.815
nb	Naive Bayes	0.5718	0.7754	0.5762	0.5869	0.5709	0.3608	0.3660	0.019
svm	SVM - Linear Kernel	0.4875	0.0000	0.4788	0.4683	0.4150	0.2233	0.2527	0.116
qda	Quadratic Discriminant Analysis	0.3764	0.5000	0.3333	0.1417	0.2059	0.0000	0.0000	0.017
dummy	Dummy Classifier	0.3764	0.5000	0.3333	0.1417	0.2059	0.0000	0.0000	0.012

Table S5- *Internal 10-fold validation of lightgbm classification algorithm (left) and hyperparameters tuning (right) results.*

Fold	Accuracy	AUC	Recall	Prec.	F1	Kappa	MCC
**0**	0.8475	0.9423	0.8471	0.8492	0.8480	0.7708	0.7711
**1**	0.8475	0.9488	0.8454	0.8527	0.8477	0.7703	0.7727
**2**	0.7627	0.9353	0.7645	0.7637	0.7615	0.6442	0.6457
**3**	0.8136	0.9446	0.8086	0.8117	0.8081	0.7191	0.7229
**4**	0.8644	0.9577	0.8625	0.8738	0.8660	0.7957	0.7990
**5**	0.7627	0.9325	0.7616	0.7697	0.7645	0.6434	0.6447
**6**	0.8475	0.9525	0.8449	0.8532	0.8479	0.7703	0.7727
**7**	0.8803	0.9663	0.8774	0.8800	0.8782	0.8201	0.8218
**8**	0.7863	0.9425	0.7863	0.7845	0.7848	0.6793	0.6798
**9**	0.8632	0.9635	0.8589	0.8669	0.8612	0.7938	0.7976
**Mean**	0.8276	0.9486	0.8257	0.8305	0.8268	0.7407	0.7428
**Std**	0.0411	0.0108	0.0401	0.0420	0.0412	0.0615	0.0620

Fold	Accuracy	AUC	Recall	Prec.	F1	Kappa	MCC
**0**	0.7881	0.9275	0.7899	0.7995	0.7890	0.6824	0.6869
**1**	0.8475	0.9348	0.8471	0.8480	0.8471	0.7707	0.7713
**2**	0.7966	0.9286	0.7967	0.7980	0.7970	0.6948	0.6951
**3**	0.7627	0.9368	0.7580	0.7584	0.7590	0.6430	0.6443
**4**	0.8051	0.9561	0.8040	0.8148	0.8070	0.7068	0.7092
**5**	0.7881	0.9332	0.7881	0.8017	0.7917	0.6825	0.6853
**6**	0.8475	0.9463	0.8452	0.8548	0.8470	0.7705	0.7741
**7**	0.8120	0.9300	0.8094	0.8110	0.8103	0.7174	0.7184
**8**	0.8034	0.9409	0.8021	0.8026	0.8024	0.7045	0.7051
**9**	0.8547	0.9638	0.8542	0.8591	0.8560	0.7814	0.7822
**Mean**	0.8106	0.9398	0.8095	0.8148	0.8106	0.7154	0.7172
**Std**	0.0287	0.0116	0.0290	0.0295	0.0291	0.0430	0.0428

3 Bayesian Network Structure Learning

3.1 Unconstrained– Python

For the development of the constrained reasoned structured network , expert judgment was applied to conditional dependencies. Some alterations of arcs include: Polydispersity index t24 and hydrodynamic size t0,t24 features were parents to exposure dose in the unconstrained structure built with the exact algorithm and no constraints in the parents relationships, however such a dependency is not realistic i.e., the external exposure dose can not be determined by the hydrodynamic size; However, the exposure dose did fed the cell viability node, which is kept in the constrained structure, eliminating the other relationships.

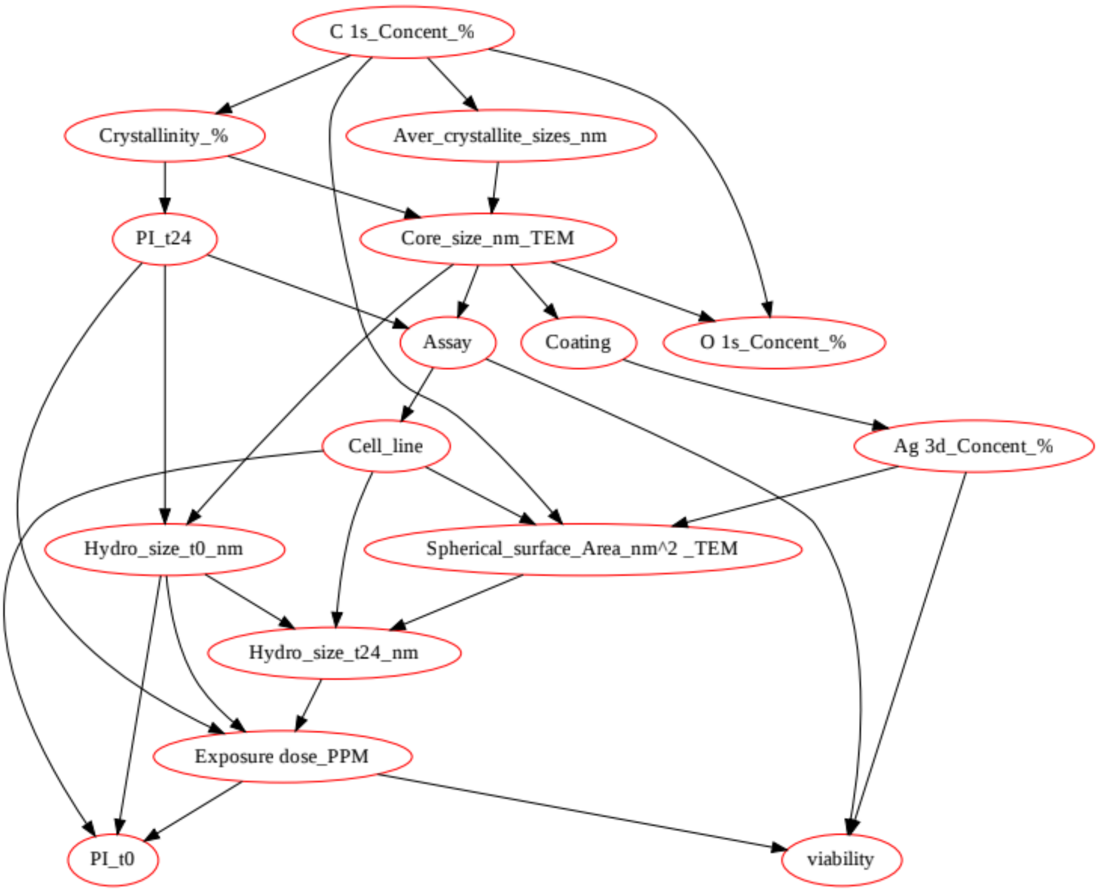

Figure S2*- Unconstrained Bayesian Network Structure.*

3.2 Unconstrained– Weka

Exploring the Weka software for automated BN construction (estimator: simple, a=0.5, search algorithm: local hill climber, six parents limit configuration), the unconstrained structure also demonstrated the exposure dose, hydrodynamic size at t24, core size and assay being connected to the outcome, reinforcing the reasoning on some arcs.

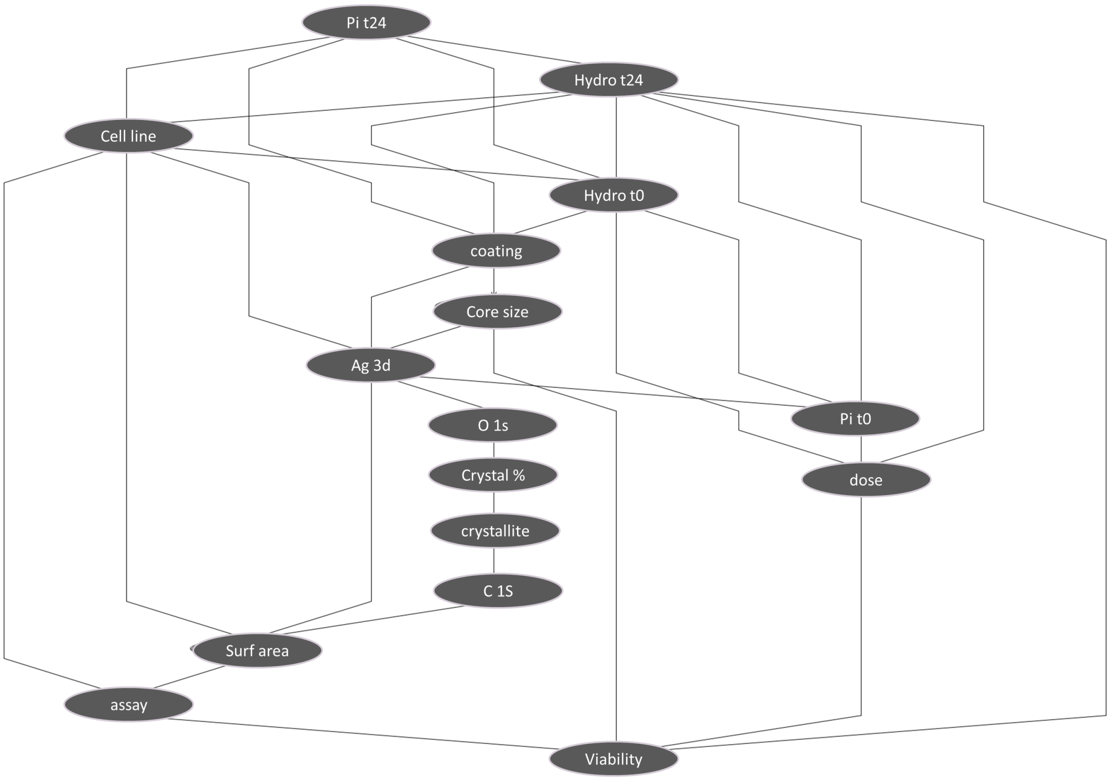

Figure S3- *Unconstrained Bayesian structure derived from WEKA software (estimator: simple, a=0.5, search algorithm: local hill climber, six parents limit configuration).*

Unconstrained Bayesian Network – Weka - Cross validation metrics

=== Stratified cross-validation === [all dataset]
Correctly Classified Instances 1324 78.7158 %
Incorrectly Classified Instances 358 21.2842 %
Total Number of Instances 1682
=== Detailed Accuracy By Class ===

	TP	FP	Prec	Rec	F-1	MCC	ROC	PRC	Class
	0.852	0.12	0.774	0.852	0.811	0.715	0.961	0.931	very_toxic
	0.854	0.131	0.785	0.854	0.818	0.711	0.946	0.903	safe
	0.645	0.07	0.809	0.645	0.718	0.615	0.914	0.848	Toxic
**Weighted** Avg.	**0.787**	**0.108**	**0.789**	**0.787**	**0.784**	**0.682**	**0.941**	**0.894**	

=== Confusion Matrix ===

```
  a   b   c   <-- classified as
 465  32  49 |   a = very_toxic
  56 516  32 |   b = safe
  80 109 343 |   c = Toxic
```

3.3 BN model Validation

Internal Cross validation metrics derived from the constrained BN structure from Python

```
              precision    recall  f1-score   support

       Toxic       0.75      0.69      0.72       427
        safe       0.80      0.79      0.79       492
  very_toxic       0.80      0.87      0.83       427

    accuracy                           0.78      1346
   macro avg       0.78      0.78      0.78      1346
weighted avg       0.78      0.78      0.78      1346


 very_toxic
Acc: 0.8665
Acc (balanced): 0.8665

 safe
Acc: 0.7886
Acc (balanced): 0.7886

 Toxic
Acc: 0.6932
Acc (balanced): 0.6932

MCC: 0.6746025781279282
```

Figure S4- *Internal cross validation (80% training data) performance metrics of the constrained BN network.*

4 Extra rules derived from the BN constrained structure

The extraction of the interpretable rules related to the quantitative intrinsic hazard properties of AgNFs, was filtered down to the cases where the hazard class was present and with the highest certainty factors (CF) as an example. The higher the CF the higher the posterior probability of that statement / rule to be true. Infinite confidence probabilities, an instance that occurs due to a divide-by-zero runtime exception when comparing the likelihood of events with no counterexamples, were discarded.

- *IF (Ag 3d atomic= High (> 15.53) ^ Hydro_size_t0_nm = Medium (209.97 -> 363.31) ^ Cell line = A549) THEN (PI_t24 = Low (0.04 -> 0.28)) 60 0.98*
- *IF (Hydro_size_t0_nm = Medium (209.97 -> 363.31) ^ PI_t24 = Medium (0.28 -> 0.499763) ^ Cell line = A549) THEN (Hydro_size t24= Medium (149.34 -> 266.9)) 18 0.94*
- *IF (Coating = HEC ^ Core size = Low (7.0 -> 17.8) ^ Hydro_size t24= Low (63.74 -> 149.34)) THEN (Spherical_surface_Area_nm^2 _TEM = Low (3981.53 -> 3981.59)) 16.69 0.94*
- *IF (Ag 3d atomic= Low (0.08 -> 0.19) ^ PI_t0 = Low (0.18 -> 0.47) ^ Cell line = A549) THEN (Hydro_size_t0_nm = Low (55.91 -> 209.97)) 13.5 0.93*
- *IF (Coating = HEC ^ PI_t24 = Medium (0.28 -> 0.49)) THEN (C 1s_Concent_% = Low (54.23 -> 60.61)) 6.19 0.86*

- *IF (Ag 3d atomic= High (> 15.53) ^ Hydro_size_t0_nm = High (> 363.315) ^ Cell line = HCT-116) THEN (PI_t24 = High (> 0.499763) 6 0.85*
- *IF (Hydro_size_t0_nm = Low (55.91 -> 209.97) ^ PI_t24 = Low (0.04 -> 0.28) ^ Cell line = A549) THEN (Hydro_size t24= Low (63.74 -> 149.34)) 5.83 0.85*
- *IF (Ag 3d atomic= High (> 15.53) ^ Hydro_size_t0_nm = Medium (209.97 -> 363.31) ^ Cell line = HCT-116) THEN (PI_t24 = Medium (0.28 -> 0.499763) 5.61 0.84*
- *IF (Ag 3d atomic= Low (0.08 -> 0.19) ^ Cell line = A549) THEN (PI_t0 = Low (0.18 -> 0.47)) 4.75 0.82*
- *IF (Ag 3d atomic= High (> 15.53) ^ Hydro_size_t0_nm = High (> 363.31) ^ Cell line = A549) THEN (PI_t24 = Low (0.04 -> 0.28)) 4 0.8*
- *IF (Ag 3d atomic= Medium (0.19 -> 15.53) ^ Hydro_size_t0_nm = High (> 363.31) ^ Cell line = A549) THEN (PI_t24 = Low (0.04 -> 0.28)) 3.69 0.78*
- *IF (Hydro_size_t0_nm = Medium (209.97 -> 363.31) ^ PI_t24 = Medium (0.28 -> 0.499763) ^ Cell line = HCT-116) THEN (Hydro_size t24= High (> 266.9)) 3.58 0.78*
- *IF (Hydro_size_t0_nm = Medium (209.97 -> 363.315) ^ PI_t24 = Low (0.04 -> 0.28) ^ Cell line = A549) THEN (Hydro_size t24= Medium (149.34 -> 266.9)) 3.53 0.77*
- *IF (Hydro_size_t0_nm = High (> 363.315) ^ PI_t24 = Medium (0.28 -> 0.49) ^ Cell line = HCT-116) THEN (Hydro_size t24= Medium (149.34 -> 266.9)) 3 0.75*
- *IF (Ag 3d atomic= Medium (0.19 -> 15.53) ^ Hydro_size_t0_nm = Medium (209.97 -> 363.315) ^ Cell line = A549) THEN (PI_t24 = Medium (0.28 -> 0.499763)) 2.88 0.74*
- *IF (Hydro_size_t0_nm = Low (55.91 -> 209.97) ^ PI_t24 = Medium (0.28 -> 0.49) ^ Cell line = A549) THEN (Hydro_size t24= Low (63.74 -> 149.34)) 2.84 0.73*
- *IF (Ag 3d atomic= Low (0.08 -> 0.19) ^ Cell line = HCT-116) THEN (PI_t0 = High (> 0.59)) 2.68 0.72826087*
- *IF (Hydro_size_t0_nm = High (> 363.315) ^ PI_t24 = Low (0.04 -> 0.28) ^ Cell line = A549) THEN (Hydro_size t24= Low (63.74 -> 149.34)) 2.43 0.70*
- *IF (Ag 3d atomic= High (> 15.53) ^ PI_t0 = High (> 0.59) ^ Cell line = HCT-116) THEN (Hydro_size_t0_nm = Medium (209.97 -> 363.315)) 2.23 0.69*
- *IF (Hydro_size_t0_nm = High (> 363.315) ^ PI_t24 = Medium (0.28 -> 0.499763) ^ Cell line = A549) THEN (Hydro_size t24= Low (63.74 -> 149.34)) 2.21 0.68*
- *IF (Ag 3d atomic= Low (0.08 -> 0.19) ^ PI_t0 = Medium (0.47 -> 0.59) ^ Cell line = HCT-116) THEN (Hydro_size_t0_nm = Low (55.91 -> 209.97)) 2.1 0.67*
- *IF (Coating = HEC ^ PI_t24 = Low (0.04 -> 0.28)) THEN (C 1s_Concent_% = Medium (60.61 -> 61.83)) 2.02 0.66*
- *IF (Ag 3d atomic= Medium (0.19 -> 15.53) ^ Cell line = HCT-116) THEN (PI_t0 = High (> 0.59)) 1.97 0.66*
- *IF (Coating = HEC ^ PI_t24 = High (> 0.49)) THEN (C 1s_Concent_% = Low (54.23 -> 60.61)) 1.94 0.65*
- *IF (Ag 3d atomic= High (> 15.53) ^ PI_t0 = Medium (0.47 -> 0.59) ^ Cell line = A549) THEN (Hydro_size_t0_nm = Medium (209.97 -> 363.315)) 1.87 0.651567944*
- *IF (Ag 3d atomic= High (> 15.53) ^ Cell line = A549) THEN (PI_t0 = Low (0.18 -> 0.47)) 1.37 0.57*

- IF (Hydro_size_t0_nm = High (> 363.315) ^ PI_t24 = High (> 0.499763) ^ Cell line = HCT-116) THEN (Hydro_size t24= High (> 266.9)) 1.33 0.57
- IF (Ag 3d atomic= Medium (0.19 -> 15.53) ^ PI_t0 = Low (0.18 -> 0.47) ^ Cell line = A549) THEN (Hydro_size_t0_nm = Medium (209.97 -> 363.315)) 1.29 0.56
- IF (Ag 3d atomic= High (> 15.53) ^ Cell line = HCT-116) THEN (PI_t0 = Medium (0.47 -> 0.59)) 1.24 0.55
- IF (Ag 3d atomic= Medium (0.19 -> 15.53) ^ PI_t0 = Medium (0.47 -> 0.59) ^ Cell line = A549) THEN (Hydro_size_t0_nm = Medium (209.97 -> 363.315)) 1.21 0.54
- IF (Ag 3d atomic= Medium (0.19 -> 15.53) ^ PI_t0 = High (> 0.59) ^ Cell line = HCT-116) THEN (Hydro_size_t0_nm = High (> 363.315)) 1.18 0.54
- IF (Ag 3d atomic= Low (0.08 -> 0.19) ^ Hydro_size_t0_nm = Low (55.91 -> 209.97) ^ Cell line = A549) THEN (PI_t24 = Low (0.04 -> 0.28)) 1.16 0.53
- IF (Crystallinity = Low (22.9 -> 60.0) ^ Core size = Low (7.0 -> 17.8) ^ Spherical_surface_Area_nm^2 _TEM = Medium (3981.59 -> 5026.55) ^ Ag 3d atomic= Low (0.08 -> 0.19) ^ Hydro_size t24= Medium (149.34 -> 266.9) ^ Exposure dose = Medium (20.0 -> 58.35) ^ Assay = WST-1) THEN (viability = safe) 35 0.97
- IF (Crystallinity = Low (22.9 -> 60.0) ^ Core size = Low (7.0 -> 17.8) ^ Spherical_surface_Area_nm^2 _TEM = Low (3981.53 -> 3981.59) ^ Ag 3d atomic= Low (0.08 -> 0.19) ^ Hydro_size t24= Low (63.74 -> 149.34) ^ Exposure dose = Low (0.0 -> 20.0) ^ Assay = MTT) THEN (viability = safe) 33 0.97
- IF (Crystallinity = Low (22.9 -> 60.0) ^ Core size = Low (7.0 -> 17.8) ^ Spherical_surface_Area_nm^2 _TEM = Medium (3981.59 -> 5026.55) ^ Ag 3d atomic= Low (0.08 -> 0.19) ^ Hydro_size t24= Medium (149.34 -> 266.9) ^ Exposure dose = Low (0.0 -> 20.0) ^ Assay = WST-1) THEN (viability = safe) 22 0.95
- IF (Crystallinity = Medium (60.0 -> 61.0) ^ Core size = Medium (17.8 -> 20.0) ^ Spherical_surface_Area_nm^2 _TEM = Medium (3981.59 -> 5026.55) ^ Ag 3d atomic= Medium (0.19 -> 15.53) ^ Hydro_size t24= High (> 266.9) ^ Exposure dose = Medium (20.0 -> 58.35) ^ Assay = WST-1) THEN (viability = Toxic) 10 0.9
- IF (Crystallinity = Low (22.9 -> 60.0) ^ Core size = High (> 20.0) ^ Spherical_surface_Area_nm^2 _TEM = High (> 5026.55) ^ Ag 3d atomic= High (> 15.53) ^ Hydro_size t24= Medium (149.34 -> 266.9) ^ Exposure dose = Low (0.0 -> 20.0) ^ Assay = MTT) THEN (viability = safe) 9.5 0.90
- IF (Crystallinity = High (> 61.0) ^ Core size = Low (7.0 -> 17.8) ^ Spherical_surface_Area_nm^2 _TEM = Low (3981.53 -> 3981.59) ^ Ag 3d atomic= Low (0.08 -> 0.19) ^ Hydro_size t24= Low (63.74 -> 149.34) ^ Exposure dose = Low (0.0 -> 20.0) ^ Assay = MTT) THEN (viability = safe) 7.75 0.88
- IF (Crystallinity = High (> 61.0) ^ Core size = Low (7.0 -> 17.8) ^ Spherical_surface_Area_nm^2 _TEM = Low (3981.53 -> 3981.59) ^ Ag 3d atomic= Low (0.08 -> 0.19) ^ Hydro_size t24= Low (63.74 -> 149.34) ^ Exposure dose = Medium (20.0 -> 58.35) ^ Assay = MTT) THEN (viability = safe) 7 0.875
- IF (Crystallinity = Medium (60.0 -> 61.0) ^ Core size = Medium (17.8 -> 20.0) ^ Spherical_surface_Area_nm^2 _TEM = Medium (3981.59 -> 5026.55) ^ Ag 3d atomic=

Medium (0.19 -> 15.53) ^ Hydro_size t24= Low (63.74 -> 149.34) ^ Exposure dose = High (> 58.35) ^ Assay = MTT) THEN (viability = Toxic) 7 0.87

- IF (Crystallinity = Low (22.9 -> 60.0) ^ Core size = Low (7.0 -> 17.8) ^ Spherical_surface_Area_nm^2 _TEM = Medium (3981.59 -> 5026.55) ^ Ag 3d atomic= Low (0.08 -> 0.19) ^ Hydro_size t24= Medium (149.34 -> 266.9) ^ Exposure dose = Low (0.0 -> 20.0) ^ Assay = Alamar Blue) THEN (viability = very_toxic) 6.67 0.86
- IF (Crystallinity = High (> 61.0) ^ Core size = Low (7.0 -> 17.8) ^ Spherical_surface_Area_nm^2 _TEM = Low (3981.53 -> 3981.59) ^ Ag 3d atomic= Low (0.08 -> 0.19) ^ Hydro_size t24= Low (63.74 -> 149.34) ^ Exposure dose = Medium (20.0 -> 58.35) ^ Assay = Alamar Blue) THEN (viability = very_toxic) 6.5 0.86
- IF (Crystallinity = Low (22.9 -> 60.0) ^ Core size = High (> 20.0) ^ Spherical_surface_Area_nm^2 _TEM = High (> 5026.55) ^ Ag 3d atomic= High (> 15.53) ^ Hydro_size t24= Medium (149.34 -> 266.9) ^ Exposure dose = Low (0.0 -> 20.0) ^ Assay = Alamar Blue) THEN (viability = very_toxic) 3.57 0.78
- IF (Crystallinity = Low (22.9 -> 60.0) ^ Core size = High (> 20.0) ^ Spherical_surface_Area_nm^2 _TEM = High (> 5026.55) ^ Ag 3d atomic= High (> 15.53) ^ Hydro_size t24= Medium (149.34 -> 266.9) ^ Exposure dose = Medium (20.0 -> 58.35) ^ Assay = Alamar Blue) THEN (viability = very_toxic) 3.5 0.77
- IF (Crystallinity = Medium (60.0 -> 61.0) ^ Core size = Medium (17.8 -> 20.0) ^ Spherical_surface_Area_nm^2 _TEM = Medium (3981.59 -> 5026.55) ^ Ag 3d atomic= Medium (0.19 -> 15.53) ^ Hydro_size t24= Low (63.74 -> 149.34) ^ Exposure dose = High (> 58.35) ^ Assay = Alamar Blue) THEN (viability = Toxic) 3.17 0.76
- IF (Crystallinity = Medium (60.0 -> 61.0) ^ Core size = Medium (17.8 -> 20.0) ^ Spherical_surface_Area_nm^2 _TEM = Medium (3981.59 -> 5026.55) ^ Ag 3d atomic= Medium (0.19 -> 15.53) ^ Hydro_size t24= Low (63.74 -> 149.34) ^ Exposure dose = Medium (20.0 -> 58.35) ^ Assay = MTT) THEN (viability = Toxic)3.12 0.75
- IF (Crystallinity = High (> 61.0) ^ Core size = Low (7.0 -> 17.8) ^ Spherical_surface_Area_nm^2 TEM = Low (3981.53 -> 3981.59) ^ Ag 3d atomic= Low (0.08 -> 0.19) ^ Hydro_size t24= Low (63.74 -> 149.34) ^ Exposure dose = High (> 58.35) ^ Assay = MTT) THEN (viability = safe) 3 0.75
- IF (Crystallinity = Medium (60.0 -> 61.0) ^ Core size = Medium (17.8 -> 20.0) ^ Spherical_surface_Area_nm^2 _TEM = Medium (3981.59 -> 5026.55) ^ Ag 3d atomic= Medium (0.19 -> 15.53) ^ Hydro_size t24= Medium (149.34 -> 266.9) ^ Exposure dose = High (> 58.35) ^ Assay = Alamar Blue) THEN (viability = Toxic) 2.75 0.733
- IF (Crystallinity = Medium (60.0 -> 61.0) ^ Core size = Medium (17.8 -> 20.0) ^ Spherical_surface_Area_nm^2 _TEM = Medium (3981.59 -> 5026.55) ^ Ag 3d atomic= Medium (0.19 -> 15.53) ^ Hydro_size t24= Medium (149.34 -> 266.9) ^ Exposure dose = Low (0.0 -> 20.0) ^ Assay = Alamar Blue) THEN (viability = safe)2.75 0.73
- IF (Crystallinity = Medium (60.0 -> 61.0) ^ Core size = Medium (17.8 -> 20.0) ^ Spherical_surface_Area_nm^2 _TEM = Medium (3981.59 -> 5026.55) ^ Ag 3d atomic= Medium (0.19 -> 15.53) ^ Hydro_size t24= Medium (149.34 -> 266.9) ^ Exposure dose = Low (0.0 -> 20.0) ^ Assay = MTT) THEN (viability = safe) 2.2 0.68

- *IF (Crystallinity = Low (22.9 -> 60.0) ^ Core size = Low (7.0 -> 17.8) ^ Spherical_surface_Area_nm^2 _TEM = Medium (3981.59 -> 5026.55) ^ Ag 3d atomic= Low (0.08 -> 0.19) ^ Hydro_size t24= Medium (149.34 -> 266.9) ^ Exposure dose = High (> 58.35) ^ Assay = WST-1) THEN (viability = safe) 2.14 0.6815*
- *IF (Crystallinity = Low (22.9 -> 60.0) ^ Core size = High (> 20.0) ^ Spherical_surface_Area_nm^2 _TEM = High (> 5026.55) ^ Ag 3d atomic= High (> 15.53) ^ Hydro_size t24= High (> 266.9) ^ Exposure dose = Medium (20.0 -> 58.35) ^ Assay = WST-1) THEN (viability = Toxic) 2.08 0.67*
- *IF (Crystallinity = Medium (60.0 -> 61.0) ^ Core size = Medium (17.8 -> 20.0) ^ Spherical_surface_Area_nm^2 _TEM = Medium (3981.59 -> 5026.55) ^ Ag 3d atomic= Medium (0.19 -> 15.53) ^ Hydro_size t24= High (> 266.9) ^ Exposure dose = Low (0.0 -> 20.0) ^ Assay = WST-1) THEN (viability = safe) 2 0.66*
